# The Role of Cerebellum and Basal Ganglia Functional Connectivity in Altered Voluntary Movement Execution in Essential Tremor

**DOI:** 10.1007/s12311-024-01699-6

**Published:** 2024-05-18

**Authors:** Massimiliano Passaretti, Claudia Piervincenzi, Viola Baione, Gabriele Pasqua, Donato Colella, Sara Pietracupa, Nikolaos Petsas, Luca Angelini, Antonio Cannavacciuolo, Giulia Paparella, Alfredo Berardelli, Patrizia Pantano, Matteo Bologna

**Affiliations:** 1 Department of Human Neurosciences, Sapienza University of Rome, Viale dell’Università, 30, 00185 Rome, Italy; 2 Department of Clinical Neuroscience, Karolinska Institutet, Stockholm, Sweden; 3 Department of Medicine, Surgery and Neuroscience, University of Siena, Siena, Italy; 4 IRCCS Neuromed, Pozzilli, IS Italy; 5 Department of Public Health and Infectious Disease, Sapienza University of Rome, Rome, Italy

**Keywords:** Essential tremor, Dentate nucleus, Globus pallidus, Resting state functional connectivity, Kinematic analysis

## Abstract

Substantial evidence highlights the role of the cerebellum in the pathophysiology of tremor in essential tremor (ET), although its potential involvement in altered movement execution in this condition remains unclear. This study aims to explore potential correlations between the cerebellum and basal ganglia functional connectivity and voluntary movement execution abnormalities in ET, objectively assessed with kinematic techniques. A total of 20 patients diagnosed with ET and 18 healthy subjects were enrolled in this study. Tremor and repetitive finger tapping were recorded using an optoelectronic kinematic system. All participants underwent comprehensive 3T-MRI examinations, including 3D-T1 and blood-oxygen-level dependent (BOLD) sequences during resting state. Morphometric analysis was conducted on the 3D-T1 images, while a seed-based analysis was performed to investigate the resting-state functional connectivity (rsFC) of dorsal and ventral portions of the dentate nucleus and the external and internal segments of the globus pallidus. Finally, potential correlations between rsFC alterations in patients and clinical as well as kinematic scores were assessed. Finger tapping movements were slower in ET than in healthy subjects. Compared to healthy subjects, patients with ET exhibited altered FC of both dentate and globus pallidus with cerebellar, basal ganglia, and cortical areas. Interestingly, both dentate and pallidal FC exhibited positive correlations with movement velocity in patients, differently from that we observed in healthy subjects, indicating the higher the FC, the faster the finger tapping. The findings of this study indicate the possible role of both cerebellum and basal ganglia in the pathophysiology of altered voluntary movement execution in patients with ET.

**Supplementary Information:**

The online version contains supplementary material available at 10.1007/s12311-024-01699-6.

## Introduction

Essential tremor (ET) is a neurological condition clinically characterized by bilateral action tremor of the upper limb lasting for at least 3 years [1]. Previous neurophysiological studies have demonstrated an altered voluntary movement execution, manifested as movement slowness (bradykinesia) in a significant proportion of ET patients [2–7].

It is widely acknowledged that dysfunction within the cerebello-thalamo-cortical circuit plays a significant role in the pathophysiology of ET [8–12]. Resting-state functional magnetic resonance imaging (rs-fMRI) studies have consistently demonstrated impaired functional connectivity (FC) between the cerebellum and both cortical and subcortical regions in ET patients [13–17]. The dentate nucleus (DN), which is the primary cerebellar output, projects to the thalamus and cortical areas. DN is composed by dorsal (motor) and ventral (associative) domains [18]. The dorsal domain receives projection from the lateral cerebellum and is connected via ventro-lateral nuclei of thalamus with motor cortical areas (motor cortex, premotor cortex, supplementary motor area, somatosensory cortex) whereas the ventral domain is connected via dorsomedial thalamus nuclei with associative cortices (prefrontal cortex, intraparietal and inferior parietal cortex) [19–21]. Both domains are connected with striatum via intralaminar nuclei of thalamus [22]. Alterations in DN-FC, both within and outside the cerebellum, is present in ET [9]. Tikoo et al., found a correlation between DN functional disconnection and the severity of tremor as well as cognitive impairment in ET patients [17]. Given the key role of the cerebellum in ET pathophysiology, it is plausible that DN dysfunction may also play a role in the pathophysiology of altered voluntary movement execution in ET patients [2, 23, 24]. This hypothesis is supported by the observation that various kinematic parameters, particularly movement direction and velocity, are encoded in cerebellar neural activity. In particular, the spike firing of Purkinje cells, the main output of the cerebellar cortex, is thought to control movement velocity across multiple tasks [2]. Furthermore, several studies have shown that degenerative cerebellar disease, cerebellar tumors and ischemic lesions may be associated with slowed movement execution (bradykinesia) [7, 25]. Additional mechanisms, however, may involve a wider cerebral network, including not only the cerebellum and interconnected cortical motor areas but also the basal ganglia (BG). In this regard, the globus pallidus (GP) serves as the central output hub within the BG circuitry, essential for efficient and fast voluntary movement execution [26, 27]. GP is distinct in two parts, the internal (GPi) is deputed of motor output of BG; and the external (GPe) is a regulatory relay in BG circuit, classically acknowledged in the indirect pathway [28]. GP has been implicated in fine regulation of movement velocity and amplitude, regulating frequency and synchronicity of oscillation in motor network [29–31]. ET patients demonstrated reduced structural connectivity within the GP, caudate, and supplementary motor area (SMA) [32]. Hence, together with the cerebellum, the BG may also contribute to the pathophysiology of altered voluntary movement execution in ET, as recently proposed [33].

To the best of our knowledge, there is no research study investigating the relationship between quantitative kinematic measurements of voluntary movement execution, and the activity of brain networks in individuals with ET. Moreover, no previous studies have thoroughly examined possible changes in connectivity within (or between) the cerebellum and BG, focusing on GP network activity as main voluntary movement BG output, and their potential implications in ET pathophysiology. Therefore, the primary objective of this study is to investigate the morphometric and functional changes of the cerebellum and BG in ET patients and to explore the correlations between these changes and kinematic measures of altered movement execution observed during repetitive finger tapping tasks, which are widely employed in clinical practice to evaluate bradykinesia [2, 34]. By conducting this study, we aim to gain deeper insights into the role of the cerebellum and BG in the pathophysiology of movement execution alterations in ET.

## Methods

### Participants and Clinical Assessment

This study included 20 ET patients (3 females) with a mean age of 67.7 ± 13.4 years and a disease duration of 13.9 ± 9.9 years. ET patients were consecutively enrolled from the Movement Disorder Outpatient Clinic at the Department of Human Neurosciences, Sapienza University of Rome, Italy. The control group consisted of 18 healthy subjects (HS) (6 females) with a mean age of 62.8 ± 7.8 years. The clinical diagnosis of ET was based on the established clinical diagnostic criteria [1]. ET patients with clinically detectable soft-signs (ET-plus patients with rest tremor, questionable dystonia and ataxia) and psychiatric conditions were excluded from the study. Clinical evaluations of the patients were conducted by a neurologist with expertise in movement disorders (MB). To minimize any potential confounding effects of medication, all patients discontinued their medications 48 hours prior to the experiment. Five out of 20 subjects exhibited head tremor, yet this did not lead to significant artifacts, as also demonstrated by ICA-AROMA analysis. Clinical assessment of ET patients was performed using the Fahn-Tolosa-Marin Tremor Rating Scale (FTMTRS) [35] and the Movement Disorder Society -sponsored revision of the Unified Parkinson’s Disease Rating Scale, motor section (MDS-UPDRS-III) [36]. All participants also underwent the Montreal Cognitive Assessment (MoCA) [37] and the Beck Depression Inventory (BDI-II) [38] to define the cognitive profile and depressive symptoms. Study participants underwent the kinematic evaluation and MRI session on two separate days, within one week. All enrolled subjects provided written informed consent for the use of their data for research purposes. The study protocol was approved by the institutional ethics committee and conducted in accordance with the principles outlined in the Declaration of Helsinki.

### Kinematic Recordings and Analysis

The kinematic assessment was conducted using an optoelectronic system (SMART motion system, BTS Engineering, Italy), which consisted of three infrared cameras operating at a frequency of 120 Hz. Reflective markers of negligible weight were attached to the participant's hand, with three markers placed at the wrist and two markers on the distal phalanx of the thumb and index finger to evaluate finger tapping movements. Participants were comfortably seated in a chair and instructed to perform ‘as wide and fast as possible’ repetitive opposition movement of the thumb and index finger (finger tapping). Each trial lasted for 15 seconds, and the task was repeated three times for each hand. A rest interval of 60 seconds was provided between trials to prevent fatigue [2, 34, 39].

The kinematic recordings were analyzed using dedicated software (SMART Analyzer, BTS, Milan, Italy). This software utilized an automatized algorithm and linear regression techniques to calculate the relevant kinematic variables, including the number of movements, amplitude (in degrees), velocity (in degrees per second), as well as amplitude and velocity decrement during 15 seconds of repetitive finger movements. Additionally, the coefficient of variation (CV) was also measured to quantify movement rhythm. The CV was calculated by dividing the standard deviation by the mean value of the inter-tap intervals, with higher CV values indicating less rhythmic repetitive movements [2, 34, 39]. In ET, we performed also postural tremor analysis. To this aim, we used two markers placed on each hand. Three 45-sec recordings of postural tremor were obtained with the upper limbs positioned forward to the chest [2, 40–43]. Tremor analysis was performed using the same dedicated software (SMART Analyzer, BTS Engineering, Italy). The magnitude of tremor was analyzed by measuring the root-mean-square (RMS) of the acceleration traces of the reference marker (on the second metacarpal bone) in 3D space and then was expressed in GRMS^2. Power spectra were quantified by means of fast Fourier transformation [2, 40–43]. We then measured the dominant frequency peak (Hz) of postural tremor.

### MRI Acquisition

Participants underwent a multimodal 3T-MRI scan using a 12-channel head coil for parallel imaging (Verio, Siemens AG). The MRI protocol included a high-resolution 3-dimensional T1-weighted MPRAGE (3D T1) sequence with 176 contiguous sagittal slices, 1-mm thick (TR = 1900 ms, TE = 2.93 ms, flip angle = 9˚, matrix = 256 × 256, FOV = 260 mm^2). T2-weighted images were also acquired (TR = 3320 ms, TE = 10/103 ms, FOV = 220 mm^2, 384 × 384 matrix, 25 4-mm thick slices, 30% gap). Additionally, blood oxygenation level-dependent (BOLD) single-shot echo-planar images were acquired (TR = 3000 ms, TE = 30 ms, flip angle = 89˚, 64 × 64 matrix, 50 slices, 140 volumes, acquisition time = 7 min, voxel size 3 mm^3). During the MRI scan, participants were instructed to lie down with their eyes closed and remain awake.

### MRI Analysis

Structural and functional data were pre-processed using FMRIB’s Software Library (FSL), version 6.0.1 (http://fsl.fmrib.ox.ac.uk/fsl).

#### Structural MRI

Three-dimensional (3D) T1-weighted images were skull stripped using FSL’s Brain Extraction Tool (BET) followed by segmentation into grey matter (GM) white matter (WM) and cerebrospinal fluid (CSF) via FMRIB automated segmentation tool (FAST). Brain tissue volumes, normalized for head size, were estimated with SIENAX [44]. Volumes of subcortical grey matter structures were calculated by FMRIB’s Integrated Registration and Segmentation Tool (FIRST), part of FSL (http://fsl.fmrib.ox.ac.uk/fsl/fslwiki/FIRST).

To calculate cerebellar volume on the 3D T1 images, we used the spatially unbiased infratentorial template toolbox (SUIT), version 3.4 (http://www.diedrichsenlab.org/imaging.suit.htm), implemented in SPM12 (http://www.fil.ion.ucl.ac.uk/spm) running under MATLAB R2020. The following steps were performed with SUIT: 1) extraction of each subject's cerebellum from 3DT1 anatomical images; 2) normalization of the isolated cerebellum to the SUIT atlas template space using the affine transformation matrix and non-linear flow field; in particular, in the present work the *suit_normalize_dentate* module of SUIT normalization was used to ensure accurate individual isolation of the DN 3) re-slicing of the cerebellum, in order to preserve the volume of cerebellar lobules in the SUIT atlas template space. Lastly, the obtained SUIT atlas was realigned back to the native subject space. For each subject, we parceled and computed 28 cerebellar lobules and 6 nuclei (dentate, interposed and fastigial). Left and right cerebellar volumes were computed as the sum of lobules I–IV, V, VI, Crus I, Crus II, VIIa, VIIIa, VIIIb, IX, and X and used for further statistical analyses. Left and right dentate volumes were also extracted.

#### Functional MRI

Preprocessing of functional data included the following steps: removal of the first three volumes to allow the signal to reach equilibrium; spatial smoothing at 4 mm full width at half maximum Gaussian kernel; movement removal with independent component analysis- automatic removal of motion artifacts (ICA-AROMA) [45] application of a band-pass filter [0.008-0.09 Hz] to exclude physiological artifacts; and further movement and artifact correction via WM and CSF signal regression. A two-step procedure for the linear/non-linear registration of subject functional images on standard space was implemented using FMRIB’s linear image registration tool (FLIRT) and FMRIB’s non-linear registration tool (FNIRT).

#### Seed Description

For seed-based analyses, regions of interest (ROIs) were created using 2-mm-radius spheres centered on reference MNI coordinates. For DN, dorsal and ventral seeds, corresponding to motor and non-motor functional territories, were defined following previous studies [46, 47] (right dorsal: x= 12, y= -57, z= -30; left dorsal: x= -12, y= -57, z= -30; right ventral: x= 17, y= -65, z= -35; left ventral: x= -17, y= -65, z= -35).

For the GP, GPe and GPi seeds, corresponding to the inhibitory indirect and excitatory direct pathways, were defined according to Tarcijonas et al. [48] (right GPe: x= 17, y= 4, z= 1; left GPe: x= −15, y= 4, z= −4; right GPi: x= 15, y= −1, z= −2; left GPi: x= −17, y= −3, z= −4).

For each subject, left and right dorsal and ventral DN as well as left and right GPe and GPi were combined in bilateral masks and transformed into the functional space using both linear and non-linear deformations. Seed-based analyses were performed using FSL’s FMRI Expert Analysis Tool (FEAT). For each subject, the mean time series was calculated within each of the selected 4 ROIs and used as seeds in the analyses. Voxel-wise maps of FC were calculated between each seed and the rest of the brain for each individual participant via a general linear model (GLM).

### Statistical analysis

Statistical analysis was performed using SPSS software (IBM SPSS Statistics, Version 25.0. Armonk, NY: IBM Corp.). Age was compared using the Mann-Whitney U test, while gender was compared using the Fisher's exact test between the patient and control groups.

#### Kinematic data

In a preliminary analysis, paired sample t-tests were used to compare the kinematic variables of tremor and finger tapping movements of two sides of the body in both groups (ET and HS). After demonstrating no difference between sides in either group, we calculated the averages of kinematic variables of both sides and used them for group comparison by unpaired sample t-tests. The Bonferroni correction, was to account for multiple comparisons when conducting t-tests, thereby minimizing the risk of type I errors.

#### MRI Structural data

Group differences in terms of GM and WM volumes, volumes of deep grey matter structures, left and right cerebellar volumes and DN were tested via non-parametric test (Mann-Whitney U test), Bonferroni corrected (21-ROIs; corrected alpha level = 0.002).

#### MRI Functional data

Maps of dorsal and ventral DN- and GPe and GPi-FC were assessed voxel-wise in the single groups of patients and HS (one-sample t-test), and between groups (two sample unpaired t-test), via non-parametric tests (FSL randomise, 5000 permutations), including age, sex, and GM volume as covariates of no interest.

Results were corrected using false discovery rate (FDR) correction [49] for multiple comparisons (*p* < 0.05). The minimum cluster extent was set at 100 voxels.

### Correlation between kinematic and MRI data

In both HS and ET patients’ groups, voxel-wise correlations between either dorsal and ventral DN- or GPe and GPi-FC maps and finger tapping velocity were non parametrically performed (FSL randomise, 5000 permutations), with age and gender as covariates of no interest. For ET patients we also included tremor severity (GRMS^2) as covariate of no interest. Results were corrected using FDR correction [49] for multiple comparisons (*p* < 0.05). The minimum cluster extent was set at 100 voxels.

As supplementary analyses, we also performed voxel-wise correlations between dorsal and ventral DN and GPe and GPi FC maps and essential tremor severity (see Supplementary Materials for further details).

## Results

### Clinical and demographic data

There were no significant differences between ET patients and HS in terms of age (p=0.10), gender distribution (p=0.71), and MOCA scores (p=0.11). The demographic and clinical characteristics of the subjects are presented in Table 1.

**Table1:** Demographic and clinical characteristics of patients with essential tremor (ET) and healthy subjects (HS)

	ET	HS	*p*- value
Age, years	67.7 (13.4)	62.8 (7.8)	0.10
Gender, Female/Male	3/17 (20)	6/12 (18)	0.71
Disease duration, years	13.9 (9.9)	NA	NA
MOCA	26.4 (2.1)	27.7 (2)	0.11
Tremor severity (FMTRS)	0.38 (0.20)	NA	NA
MDS-UPDRS III	6.2 (3)	NA	NA

The data are presented as mean (standard deviation). MOCA refers to the Montreal Cognitive Assessment, FMTRS refers to the Fahn–Tolosa–Marin Tremor Rating Scale, and MDS-UPDRS III refers to the Movement Disorder Society Unified Parkinson's Disease Rating Scale. NA stands for not applicable.

In the ET group, the FTMTRS score was 17.7 ± 10.8, and the MDS-UPDRS-III score was 6.2 ± 3.0, with the main influence coming from the postural and kinetic tremor components (as assessed by items 3.14 and 3.15). However, none of the patients exhibited other clinically-detectable symptoms that would warrant an alternative diagnosis [50].

### Kinematic Data

ET subjects had postural upper limb tremor characterized by an average frequency of 6.07 ± 1.22 Hz and an amplitude of 0.38 ± 0.21 GRMS^2. The analysis of finger tapping revealed that ET patients had a reduced number of movements and lower velocity in their finger tapping movements compared to HS (number of movements: 41.00 ± 13.36 vs. 54.63 ± 13.76, p<0.01; velocity: 951.51 ± 187.51 vs. 1126.35 ± 142.92, p<0.01). The analysis of finger tapping movements also indicated a tendency for ET patients to exhibit lower amplitude compared to HS (amplitude: 47.49 ± 8.61 vs. 53.41 ± 8.05, p=0.04. However, it is important to note that this difference did not reach statistical significance after applying the Bonferroni correction for multiple comparisons. Finally, no significant differences were observed in terms of rhythm, and amplitude and velocity slope (sequence effect) between ET and HS (p>0.05) (Table 2).

**Table 2:** Kinematic variables of finger tapping in patients with essential tremor (ET) and healthy subjects (HS)

	ET	HS	*p*-value
N° Movements	41.00 (13.36)	54.65 (13.76)	**0.0043**
Rhythm	0.12 (0.05)	0.09 (0.09)	>0.05
Amplitude	47.49 (8.61)	53.41 (8.05)	0.0389
Amplitude slope	-0.12 (0.16)	-0.15 (0.11)	>0.05
Velocity	951.51 (187.51)	1126.35 (142.92)	**0.0034**
Velocity slope	-5.36 (2.94)	-5.57 (2.97)	>0.05

The amplitude is expressed in degrees. The amplitude slope is expressed in degrees per number of movements. The speed is expressed in degrees per second. The speed slope is expressed in degrees per second per number of movements. The rhythm is expressed as the coefficient of variation of the inter-tap intervals. The data are presented as means (standard deviation). Significant p values are in bold.

### MRI

In terms of structural MRI findings, no significant differences were observed in the volumes of whole brain GM and WM, cerebellum, and deep gray matter structures between ET patients and HS (p>0.05) (Table 3).

**Table 3:** Volumes of brain structures in patients with essential tremor (ET) and healthy subjects (HS)

	ET	HS	*p*-value
Whole GM [ml]	640.70 (52.6)	642.33 (54.3)	0.99
Whole WM [ml]	723.38 (64.5)	704.49 (66.6)	0.58
Left Pallidum [ml]	1.56 (0.22)	1.72 (0.15)	0.05
Right Pallidum [ml]	1.58 (0.28)	1.70 (0.17)	0.09
Left Dentate nucleus [ml]	1.07 (0.32)	1.09 (0.31)	0.78
Right Dentate nucleus [ml]	1.26 (0.47)	1.20 (0.35)	0.87
Left Cerebellum [ml]	60.93 (9.69)	63.18 (5.97)	0.32
Right Cerebellum [ml]	58.73 (9.61)	60.78 (5.97)	0.33

The data are presented as means (standard deviation). GM refers to Gray Matter, and WM refers to White Matter. To assess differences between groups in terms of GM and WM volumes, volumes of deep grey matter structures, DN volumes and left and right cerebellar volumes, non-parametric tests (Mann-Whitney U test) with Bonferroni correction were performed.

Maps of dorsal and ventral DN- and GPe and GPi-FC in the single groups of patients and HS were reported in the Supplementary Materials (Suppl. Fig. 1, Suppl. Fig.2, Suppl. Table 1 and Suppl. Table 2).

#### DN

**Fig. 1:**
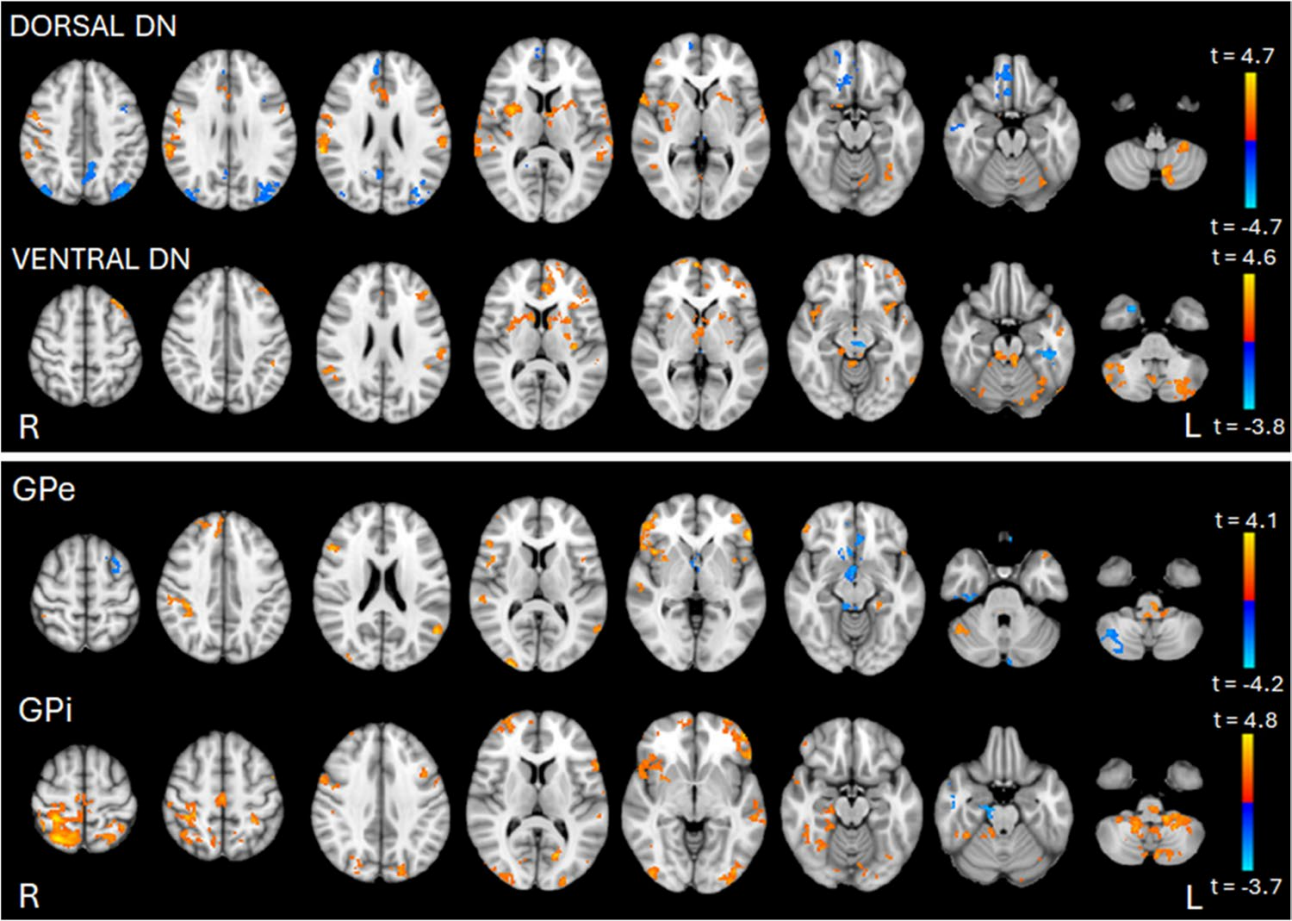
Resting-state functional connectivity (rsFC) maps of dorsal and ventral portions of the dentate nucleus (DN) and external and internal segments of the globus pallidus (GPe and GPi) comparing essential tremor (ET) patients with healthy subjects (HS). The color bar represents the *t* statistic of FC differences between the two groups. Red-yellow areas indicate where FC was significantly higher in ET patients than in HS, and blue-light blue areas indicate where FC was significantly lower in ET patients than in HS. Statistical significance was considered at p<0.05, False Discovery Rate (FDR) corrected

In the between group comparison, ET patients, compared to HS, exhibit higher FC (*p* < 0.05 FDR corrected) between dorsal DN and several regions, including cerebellum (left lobules VI, VIIb, VIIIa, VIIIb, crus I and II, right lobules I-IV, V and vermis), thalamus and BG, bilateral pre and post-central gyri and bilateral temporal and insular cortices. Additionally, they demonstrate lower FC between dorsal DN and precuneus, right inferior and middle temporal gyri, parieto-occipital cortices and orbitofrontal regions (Fig. 1, Table 4).

**Table 4:** Brain regions showing significant dorsal and ventral dentate nucleus (DN) functional connectivity differences between patients with essential tremor (ET) and healthy subjects (HS) (*p* <0.05, false discovery rate corrected, minimum cluster extent set at 100 voxels). Anatomical localizations of peak MNI coordinates were established according to Harvard-Oxford cortical and subcortical structural atlases and the cerebellar atlas included in FMRIB’s Software Library

Cluster size (voxels)	MNI coordinates				Cluster location (local maxima)
	T	x	y	z	
Dorsal DN FC					
ET > HS					
1231	4.46	62	-24	20	R Parietal Operculum Cortex
	4.24	50	-2	32	R Precentral Gyrus
	3.96	66	-24	14	R Planum Temporale
	3.64	64	-38	16	R Supramarginal Gyrus
	3.17	62	-12	16	R Central Opercular Cortex
772	4.66	32	2	8	R Putamen
	4.03	36	4	12	R Central Opercular Cortex
	3.49	38	-4	-8	R Insular Cortex
	3.36	46	-18	-4	R Superior Temporal Gyrus, posterior division
	2.56	26	-4	-10	R Amygdala
503	3.30	-62	-20	18	L Postcentral Gyrus
	3.30	-62	0	6	L Precentral Gyrus
	3.12	-68	-24	8	L Superior Temporal Gyrus
	3.10	-58	-26	22	L Supramarginal Gyrus, anterior division
	2.92	-58	-22	18	L Central Opercular Cortex
	2.90	-62	-14	6	L Planum Temporale
	2.71	-56	-42	12	L Supramarginal Gyrus, posterior division
	2.51	-52	-26	14	L Parietal Operculum Cortex
293	3.68	-12	-64	-52	L Cerebellar Lobule VIIIa
	3.09	-16	-76	-50	L Cerebellar Lobule VIIb
	2.47	-24	-46	-52	L Cerebellar Lobule VIIIb
	2.42	-34	-76	-38	L Cerebellar Crus II
	2.29	-28	-70	-36	L Cerebellar Crus I
219	3.55	-4	-2	6	L Thalamus
	2.81	-20	14	0	L Putamen
	2.60	-30	8	12	L Insular Cortex
	2.22	-10	4	10	L Caudate
	2.02	2	-6	8	R Thalamus
215	3.08	-2	-66	-2	L Lingual Gyrus
	2.69	2	-60	-8	R Cerebellar Lobule V
	2.67	-8	-66	-12	L Cerebellar Lobule VI
	2.01	-4	-72	-18	Vermis Lobule VI
177	3.27	-52	-2	-6	L Planum Polare
	3.16	-38	-10	-4	L Insular Cortex
	2.76	-50	-10	-6	L Superior Temporal Gyrus, anterior division
	2.75	-52	8	-2	L Temporal Pole
	1.98	-48	6	-2	L Central Opercular Cortex
174	3.36	-36	-58	-12	L Temporal Occipital Fusiform Cortex
	3.20	-34	-66	-18	L Occipital Fusiform Gyrus
	2.93	-28	-62	-18	L Cerebellar Lobule VI
	2.83	-44	-58	0	L Middle Temporal Gyrus, temporooccipital part
158	3.07	48	-54	4	R Middle Temporal Gyrus, temporooccipital part
	2.86	62	-62	-6	R Lateral Occipital Cortex, inferior division
	2.48	46	-54	-6	R Inferior Temporal Gyrus, temporooccipital part
	2.44	36	-64	-12	R Occipital Fusiform Gyrus
127	2.71	22	-30	-30	R Cerebellar Lobule I-IV
	2.70	22	-32	-34	Brainstem
	2.69	22	-26	-28	R Parahippocampal Gyrus, posterior division
	2.59	28	-36	-32	R Cerebellar Lobule V
116	3.93	56	4	6	R Central Opercular Cortex
	2.57	58	6	-2	R Planum Polare
112	3.23	20	-34	68	R Postcentral Gyrus
	3.10	18	-28	66	R Precentral Gyrus
111	3.18	44	44	0	R Frontal Pole
108	3.20	6	-20	-36	Brainstem
103	2.81	10	26	26	R Cingulate Cortex, anterior division
	1.97	8	34	26	R Paracingulate Gyrus, anterior division
HS > ET					
868	3.62	-40	-72	38	L Lateral Occipital Cortex, superior division
452	4.42	32	-68	54	R Lateral Occipital Cortex, superior division
360	3.02	-6	-62	36	L Precuneous Cortex
	2.38	6	-60	22	R Precuneous Cortex
302	4.01	56	-8	-28	R Middle Temporal Gyrus, posterior division
	2.71	50	-10	-34	R Inferior Temporal Gyrus, posterior division
	2.56	60	0	-30	R Middle Temporal Gyrus, anterior division
245	2.88	4	28	-22	R Subcallosal Cortex
	2.86	14	42	-18	R Frontal Pole
	2.68	10	32	-18	R Frontal Medial Cortex
	2.64	14	24	-14	R Frontal Orbital Cortex
187	3.02	12	68	18	R Frontal Pole
	3.00	8	48	22	R Paracingulate Gyrus
	2.76	6	54	22	R Superior Frontal Gyrus
145	3.12	-36	2	36	L Middle Frontal Gyrus
113	2.69	28	-60	58	R Lateral Occipital Cortex, superior division
	2.63	40	-50	54	R Superior Parietal Lobule
103	3.82	26	28	48	R Middle Frontal Gyrus
VENTRAL DN FC					
ET > HS					
1680	4.02	-2	34	16	L Cingulate Gyrus, anterior division
	3.81	-42	22	50	L Middle Frontal Gyrus
	3.62	4	66	4	R Frontal Pole
	3.37	-36	44	-8	L Frontal Pole
1177	3.39	-24	-68	-40	L Cerebellar Crus II
	3.29	-32	-74	-38	L Cerebellar Crus I
	3.28	-28	-52	-24	L Cerebellar Lobule VI
	3.14	0	-64	-48	Vermis Lobule VIIIb
	2.84	-24	-72	-46	L Cerebellar Lobule VIIb
825	3.37	34	-84	-38	R Cerebellar Crus II
	3.35	34	-54	-40	R Cerebellar Crus I
	2.87	28	-68	-26	R Cerebellar Lobule VI
	2.77	30	-72	-16	R Occipital Fusiform Gyrus
366	3.09	-36	14	-12	L Insular Cortex
	2.87	-40	10	-18	L Temporal Pole
	2.87	-18	12	16	L Caudate
	2.77	-30	8	-2	L Putamen
	2.73	-40	18	-12	L Frontal Orbital Cortex
	2.67	-12	2	-4	L Pallidum
319	3.43	-6	-38	-18	Brainstem
	2.77	4	-44	-12	R Cerebellar Lobule I-IV
	2.30	18	-40	-18	R Cerebellar Lobule V
211	3.76	-66	-50	-6	L Middle Temporal Gyrus, temporooccipital part
	3.42	-58	-62	-14	L Inferior Temporal Gyrus, temporooccipital part
	3.35	-52	-66	-26	L Cerebellar Crus I
	2.72	-46	-64	-28	L Cerebellar Crus I
	2.18	-64	-38	0	L Middle Temporal Gyrus, posterior division
	1.88	-60	-42	4	L Superior Temporal Gyrus, posterior division
192	3.22	-50	-2	-6	L Planum Polare
	2.89	-38	-16	-8	L Insular Cortex
	2.74	-54	-6	-20	L Middle temporal Gyrus, anterior division
	2.48	-52	-10	-22	L Middle Temporal Gyrus, posterior division
	2.01	-48	4	-14	L Temporal Pole
188	3.03	8	8	8	R Caudate
	2.72	16	-4	16	R Thalamus
	2.37	28	4	12	R Putamen
	2.25	36	0	10	R Insular Cortex
	1.79	20	2	4	R Pallidum
166	4.50	58	-44	20	R Supramarginal Gyrus
	2.66	54	-50	20	R Angular Gyrus
132	3.23	42	8	-16	R Temporal Pole
	3.10	38	14	-10	R Insular Cortex
	2.61	44	0	-10	R Planum Polare
	2.33	32	20	-16	R Frontal Orbital Cortex
126	3.89	-36	-16	14	L Insular Cortex
	2.85	-28	-8	12	L Putamen
124	2.98	-60	-22	22	L Postcentral Gyrus
	2.89	-58	-28	14	L Parietal Operculum Cortex
	2.14	-58	-32	28	L Supramarginal Gyrus
	1.83	-56	-36	10	L Superior Temporal Gyrus, posterior division
122	3.40	16	70	0	R Frontal Pole
120	2.82	-4	-10	-4	L Thalamus
	2.73	4	-8	2	R Thalamus
	2.03	14	0	-4	R Pallidum
106	3.25	-50	-44	46	L Supramarginal Gyrus, posterior division
	2.89	-44	-40	22	L Parietal Operculum Cortex
104	2.89	26	-36	-32	R Cerebellar Lobule V
	1.99	34	-38	-34	R Cerebellar Lobule VI
HS > ET					
160	3.50	24	6	-38	R Temporal Pole
	3.40	20	4	-34	R Parahippocampal Gyrus, anterior division
121	3.69	-6	-30	-6	Brainstem
	2.80	-14	-34	-8	L Parahippocampal Gyrus, posterior division
	2.50	-18	-30	-8	L Hippocampus
111	2.68	-44	-32	-22	L Inferior Temporal Gyrus, posterior division
	2.67	-38	-30	-20	L Temporal Fusiform Cortex, posterior division

ET patients also showed higher FC when compared to HS between ventral DN and several regions including the cerebellum (left lobule VI and VIIb, right lobules I-IV, lobule V and VI, bilateral crus I and II and vermis), thalamus, BG, insular and temporal cortices, supramarginal gyri and orbitofrontal regions. Additionally, ET patients exhibited lower FC between ventral DN and brainstem, temporal regions including para-hippocampal gyri and left hippocampus (Fig. 1, Table 4).

#### GP

In the between group comparison, ET patients showed higher FC (*p* < 0.05 FDR corrected) between the GPe and several regions including the cerebellum (right lobule IX, crus I and left lobule X), brainstem, right putamen, right occipital and parietal regions, insular cortex, bilateral frontal pole and inferior frontal gyri. Additionally, ET patients exhibited lower FC, compared to HS, between GPe and the cerebellum (right lobule VIIb, crus I and II), left superior and middle frontal gyri, left frontal medial cortex and right middle and inferior temporal gyri (Fig. 1, Table 5).

**Table 5:** Brain regions showing significant external and internal segments of the globus pallidus (GPe, GPi) functional connectivity differences between patients with essential tremor (ET) and healthy subjects (HS) (*p* <0.05, false discovery rate corrected, minimum cluster extent set at 100 voxels). Anatomical localizations of peak MNI coordinates were established according to Harvard-Oxford cortical and subcortical structural atlases and the cerebellar atlas included in FMRIB’s Software Library

Cluster size (voxels)	MNI coordinates				
	T	x	y	z	Cluster location (local maxima)
GPe FC					
ET > HS					
646	3.69	50	24	14	R Inferior Frontal Gyrus, pars triangularis
	3.46	42	14	0	R Insular Cortex
	3.26	30	6	-4	R Putamen
	3.05	54	38	0	R Frontal Pole
325	3.34	34	48	34	R Frontal Pole
	2.80	8	52	28	R Superior Frontal Gyrus
	2.77	10	44	32	R Paracingulate Gyrus
299	3.91	52	-30	46	R Supramarginal Gyrus, anterior division
	2.98	36	-34	40	R Postcentral Gyrus
	2.71	36	-42	42	R Superior Parietal Lobule
	2.46	46	-36	54	R Supramarginal Gyrus, posterior division
251	3.36	6	-40	-36	Brainstem
	2.46	-20	-36	-50	L Cerebellar Lobule X
	2.35	6	-48	-48	R Cerebellar Lobule IX
197	4.06	-52	32	-4	L Inferior Frontal Gyrus, pars triangularis
	2.94	-38	52	-4	L Frontal Pole
187	3.24	-54	-62	20	L Lateral Occipital Cortex, superior division
	2.91	-52	-48	28	L Supramarginal Gyrus, posterior division
	2.76	-54	-56	14	L Angular Gyrus
	2.76	-62	-62	8	L Middle Temporal Gyrus, temporooccipital part
153	2.73	48	-52	-34	R Cerebellar Crus I
	2.33	46	-48	-26	R Temporal Occipital Fusiform Cortex
135	2.83	58	-32	6	R Superior Temporal Gyrus, posterior division
	2.21	62	-30	14	R Planum Temporale
127	2.67	-32	12	-38	L Temporal Pole
	2.36	-42	26	-20	L Frontal Orbital Cortex
119	3.26	-22	-30	-18	L Parahippocampal Gyrus, posterior division
	2.97	-24	-32	-8	L Hippocampus
108	2.95	-40	10	2	L Frontal Operculum Cortex
	2.89	-40	8	6	L Central Opercular Cortex
	2.56	-50	10	2	L Inferior Frontal Gyrus, pars opercularis
100	3.30	28	-96	12	R Occipital Pole
	2.13	34	-84	18	R Lateral Occipital Cortex
HS > ET					
935	4.12	16	18	-20	R Frontal Orbital Cortex
	3.64	-2	6	-8	L Subcallosal Cortex
	3.39	-8	38	-28	L Frontal Pole
	3.12	14	50	-20	R Frontal Pole
	2.90	0	24	-22	R Subcallosal Cortex
	2.86	-10	32	-22	L Frontal Medial Cortex
185	2.99	34	-66	-50	R Cerebellar Lobule VIIb
	2.72	32	-74	-48	R Cerebellar Crus II
	2.26	52	-68	-40	R Cerebellar Crus I
185	3.44	36	-24	-26	R Temporal Fusiform Cortex, posterior division
	2.99	30	-22	-26	R Parahippocampal Gyrus, anterior division
	2.50	52	-8	-26	R Middle Temporal Gyrus, anterior division
	2.49	44	-30	-24	R Inferior Temporal Gyrus, posterior division
142	3.45	12	-32	-16	Brainstem
	1.96	10	-40	-4	R Lingual Gyrus
120	3.19	-6	-84	-40	L Cerebellar Crus I
	2.40	-4	-86	-28	L Cerebellar Crus II
112	2.87	-26	4	56	L Superior Frontal Gyrus
	2.64	-30	14	56	L Middle Frontal Gyrus
GPi FC					
ET > HS					
3639	4.75	30	-40	60	R Superior Parietal Lobule
	4.69	22	-62	60	R Lateral Occipital Cortex, superior division
	3.72	16	-44	72	R Postcentral Gyrus
	3.51	10	-56	62	R Precuneous Cortex
	3.36	40	-84	-2	R Lateral Occipital Cortex, inferior division
2271	4.35	-36	-64	-58	L Cerebellar Lobule VIIb
	4.30	10	-26	-40	Brainstem
	4.22	6	-72	-50	R Cerebellar Lobule VIIb
	4.13	10	-46	-50	R Cerebellar Lobule IX
	3.66	-24	-48	-46	L Cerebellar Lobule VIIIb
	3.65	16	-50	-56	R Cerebellar Lobule VIIIb
	3.61	-16	-40	-46	L Cerebellar Lobule X
	3.39	-18	-66	-60	L Cerebellar Lobule VIIIa
683	3.19	6	60	4	R Frontal Pole
523	4.14	-44	44	-2	L Frontal Pole
	3.65	-54	30	-2	L Inferior Frontal Gyrus, pars triangularis
	3.44	-56	16	4	L Inferior Frontal Gyrus, pars opercularis
	2.62	-52	38	2	L Frontal Pole
466	3.50	-38	-84	-4	L Lateral Occipital Cortex, inferior division
	3.08	-34	-94	-4	L Occipital Pole
	2.92	-16	-86	-20	L Occipital Fusiform Gyrus
	2.48	-10	-78	-18	L Cerebellar Lobule VI
403	4.09	-18	-70	10	L Intracalcarine Cortex
	3.27	-16	-86	42	L Lateral Occipital Cortex, superior division
	3.11	-20	-70	18	L Cuneal Cortex
333	3.48	-20	-60	68	L Lateral Occipital Cortex, superior division
	3.28	-28	-42	54	L Superior Parietal Lobule
	2.72	-26	-38	68	L Postcentral gyrus
	2.56	-12	-54	58	L Precuneous Cortex
317	3.36	54	4	-14	R Superior Temporal Gyrus, anterior division
	3.27	54	10	-6	R Temporal Pole
	3.08	46	20	-10	R Frontal Orbital Cortex
	2.74	56	22	0	R Inferior Frontal Gyrus, pars triangularis
	2.73	40	18	-2	R Insular Cortex
	2.72	30	6	-4	R Putamen
	2.70	54	18	-2	R Inferior Frontal Gyrus, pars opercularis
	2.59	48	22	-4	R Frontal Operculum Cortex
269	3.46	-50	2	46	L Precentral Gyrus
	3.36	-44	14	40	L Middle Frontal Gyrus
	2.72	-44	12	26	L Inferior Frontal Gyrus, pars opercularis
241	3.73	-22	-52	-30	L Cerebellar Lobule VI
	3.26	-32	-60	-40	L Cerebellar Crus II
	3.25	-40	-70	-12	L Occipital Fusiform Gyrus
	2.11	-36	-58	-28	L Cerebellar Crus I
220	3.50	56	8	32	R Precentral Gyrus
	2.71	46	6	44	R Middle Frontal Gyrus
	2.07	52	14	30	R Inferior Frontal Gyrus, pars opercularis
183	3.08	-60	-10	2	L Planum Temporale
	2.76	-58	-30	-4	L Middle Temporal Gyrus, posterior division
	2.50	-54	-16	2	L Heschl's Gyrus (includes H1 and H2)
	2.38	-64	-30	0	L Superior Temporal Gyrus, posterior division
153	3.62	32	-50	-38	R Cerebellar Crus I
	2.84	30	-42	-50	R Cerebellar Lobule VIIIb
	2.76	32	-42	-44	R Cerebellar Lobule VIIIa
	2.37	38	-38	-38	R Cerebellar Lobule VI
104	3.22	-6	-56	-50	L Cerebellar Lobule IX
	2.24	2	-52	-44	R Cerebellar Lobule IX
HS > ET					
160	3.68	52	-4	-26	R Middle Temporal Gyrus, anterior division
	2.63	52	6	-42	R Temporal Pole
	2.56	46	-6	-32	R Inferior Temporal Gyrus, anterior division
	2.26	50	-16	-24	R Inferior Temporal Gyrus, posterior division
119	3.16	20	-16	-26	R Parahippocampal Gyrus, anterior division
	2.98	14	-24	-20	Brainstem

Furthermore, ET compared to HS showed higher FC between the GPi and the cerebellum (bilateral crus I, lobules VI, VIIb, VIIIa, VIIIb, IX, left crus II and lobule X), brainstem, right putamen, right temporal pole and insular cortex, precuneus, parieto-occipital regions, pre- and post-central gyri, middle and inferior frontal gyri and orbitofrontal regions. Additionally, ET exhibited lower FC between GPi and right temporal regions and brainstem (Fig. 1, Table 5).

### Correlation Between Kinematic and MRI Data

#### DN

**Fig. 2:**
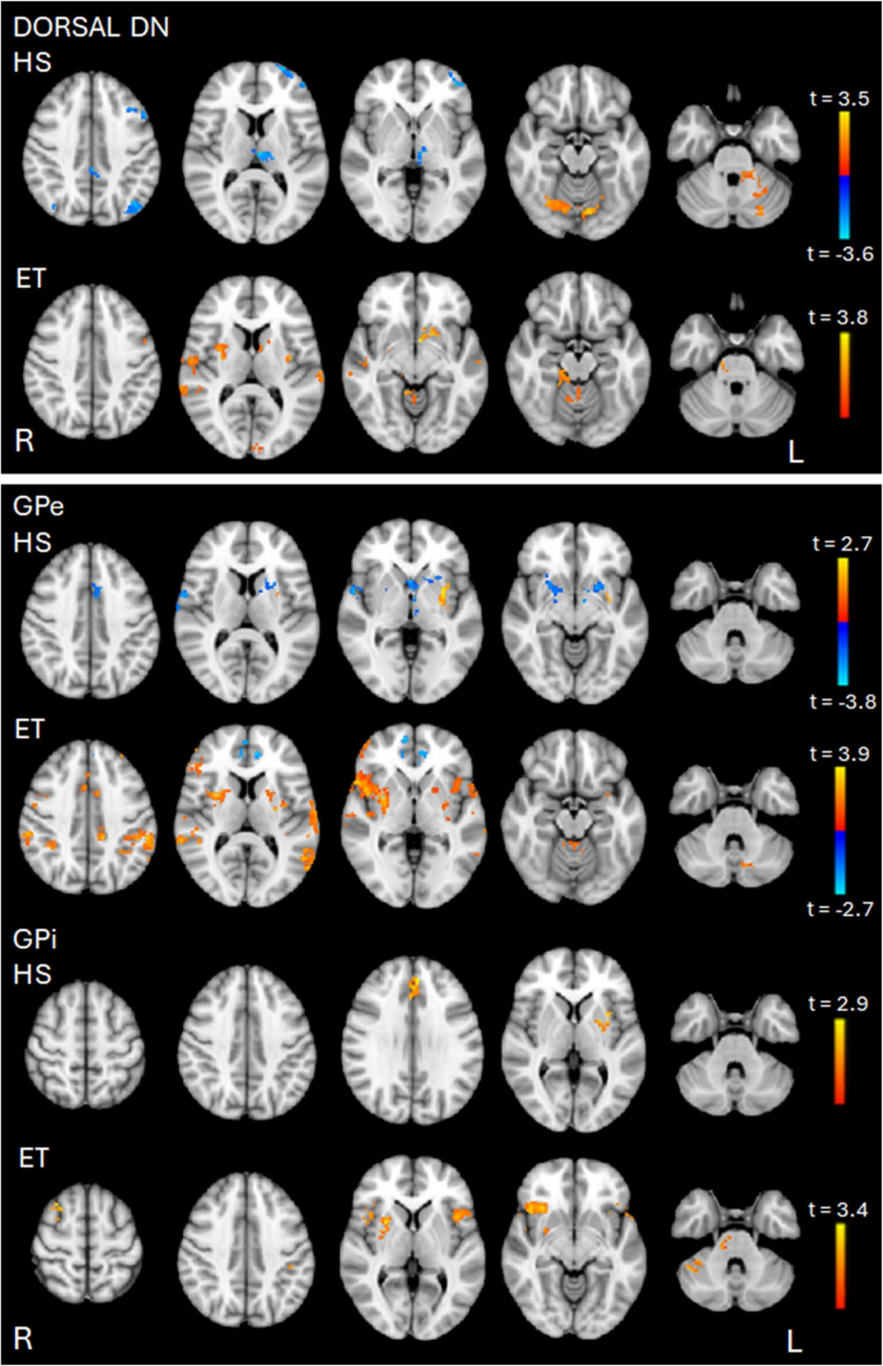
Correlation maps between velocity of finger tapping and dorsal portion of the dentate nucleus (DN) rsFC and external and internal segments of the globus pallidus (GPe and GPi) rsFC in HS and ET patients. Positive correlations are shown in red-yellow color, and negative correlations are shown in blue-light blue color. The color bar represents the *t* statistic of correlation between rsFC of the ROIs and clinical data. Significant correlation was reached if *p* < 0.05 (FDR corrected)

In HS, dorsal DN-FC with the cerebellum (lobules V-VI, left lobules I-IV, left crus I and II and vermis), brainstem and occipital fusiform cortex was positively correlated with finger tapping velocity. Instead, dorsal DN-FC with left thalamus, lateral occipital cortices, precuneus and left frontal cortex was negatively correlated with finger tapping velocity (Fig. 2, Table 6).

**Table 6:** Brain regions showing significant correlations between dorsal dentate nucleus (DN) and the external and internal segments of globus pallidus (GPe and GPi) functional connectivity maps and velocity of finger tapping, in healthy subjects (HS) and ET patients (*p* <0.05, false discovery rate corrected, minimum cluster extent set at 100 voxels). Anatomical localizations of peak MNI coordinates were established according to Harvard-Oxford cortical and subcortical structural atlases and the cerebellar atlas included in FMRIB’s Software Library

Cluster size (voxels)	MNI coordinates				Cluster location (local maxima)
	T	x	y	z	
HS Dorsal DN FC – velocity of finger tapping ↑					
276	2.77	-10	-74	-16	L Cerebellar Lobule VI
	2.16	-20	-36	-28	L Cerebellar Lobule V
	1.89	-10	-42	-28	L Cerebellar Lobule I-IV
	1.83	-10	-38	-30	Brainstem
	1.64	-6	-82	-16	L Lingual Gyrus
224	2.67	6	-68	-12	R Cerebellar Lobule VI
	2.59	30	-66	-16	R Occipital Fusiform Gyrus
	2.58	12	-60	-8	R Lingual Gyrus
	2.41	0	-70	-10	Vermis Lobule VI
	2.19	4	-62	-10	R Cerebellar Lobule V
121	3.49	-26	-78	-36	L Cerebellar Crus I
	1.80	-24	-74	-22	L Cerebellar Lobule VI
	1.52	-34	-80	-34	L Cerebellar Crus II
HS Dorsal DN FC – velocity of finger tapping ↓					
372	3.41	-34	-62	50	L Lateral Occipital Cortex
257	3.19	-20	64	16	L Frontal Pole
225	3.19	-6	-12	8	L Thalamus
179	2.70	-10	-44	38	L Cingulate Gyrus, posterior division
	1.67	-2	-44	44	L Precuneous Cortex
174	3.60	-46	18	32	L Middle Frontal Gyrus
	2.01	-40	20	22	L Inferior Frontal Gyrus, pars opercularis
164	3.04	-18	34	50	L Superior Frontal Gyrus
106	3.28	34	-72	46	R Lateral Occipital Cortex, superior division
ET Dorsal DN FC – velocity of finger tapping ↑					
354	2.77	-66	-20	12	L Superior Temporal Gyrus, posterior division
	2.54	-58	-26	18	L Parietal Operculum Cortex
	2.10	-58	-8	6	L Central Opercular Cortex
	2.09	-62	-26	24	L Supramarginal Gyrus, anterior division
	2.05	-64	-10	6	L Planum Temporale
	2.01	-62	-22	28	L Postcentral Gyrus
272	3.30	20	-38	-18	R Cerebellar Lobule V
	2.94	14	-26	-28	Brainstem
	2.75	10	-46	-8	R Cerebellar Lobule I- IV
	2.33	0	-50	-10	L Cerebellar Lobule I-IV
224	3.42	54	-20	14	R Parietal Operculum Cortex
	3.21	66	-22	24	R Supramarginal Gyrus, anterior division
	2.45	62	-18	22	R Postcentral Gyrus
	2.39	58	-16	16	R Central Opercular Cortex
	1.96	68	-18	12	R Superior Temporal Gyrus, posterior division
220	3.01	56	-40	6	R Supramarginal Gyrus, posterior division
	2.73	48	-28	0	R Superior Temporal Gyrus, posterior division
	2.39	54	-34	16	R Planum Temporale
	2.00	58	-36	-2	R Middle Temporal Gyrus, posterior division
	1.91	54	-40	-6	R Middle Temporal Gyrus, temporooccipital part
192	3.20	-18	10	-6	L Putamen
	2.88	-22	10	0	L Accumbens
	2.68	-6	0	0	L Thalamus
	1.82	-8	2	10	L Caudate
140	2.53	-10	-98	2	L Occipital Pole
138	2.85	26	-6	0	R Pallidum
	2.70	26	6	12	R Putamen
	2.03	34	-16	4	R Insular Cortex
130	3.06	-52	4	28	L Precentral Gyrus
	2.29	-54	10	24	L Inferior Frontal Gyrus
	1.83	-50	18	30	L Middle Frontal Gyrus
122	3.37	-44	-30	6	L Planum Temporale
	2.94	-44	-24	6	L Heschl's Gyrus (includes H1 and H2)
	2.91	-36	-8	8	L Insular Cortex
	2.68	-28	-14	8	L Putamen
118	3.20	68	-28	0	R Superior Temporal Gyrus, posterior division
	2.36	70	-42	4	R Middle Temporal Gyrus, temporooccipital part
	2.16	64	-38	8	R Supramarginal Gyrus, posterior division
HS GPe FC – velocity of finger tapping ↑					
175	2.63	-32	0	-6	L Putamen
	2.20	-34	-10	6	L Insular Cortex
HS GPe FC – velocity of finger tapping ↓					
476	3.75	-20	16	8	L Caudate
	2.72	-10	2	-6	L Pallidum
	2.48	0	12	0	Subcallosal Cortex
	2.04	-6	-20	6	L Thalamus
329	2.91	-4	16	44	L Paracingulate Gyrus
	2.85	2	8	60	R Juxtapositional Lobule Cortex (formerly Supplementary Motor Cortex)
	2.60	2	18	58	R Superior Frontal Gyrus
193	2.77	56	-2	4	R Central Opercular Cortex
	2.50	62	2	10	R Precentral Gyrus
	2.10	66	-4	4	R Superior Temporal Gyrus, anterior division
	2.04	66	-12	8	R Planum Temporale
	1.96	52	-4	2	R Planum Polare
178	2.66	24	8	-12	R Putamen
	2.54	20	-6	-10	R Amygdala
	2.27	18	-16	-14	R Hippocampus
	1.80	10	8	-6	R Accumbens
	1.74	28	18	-10	R Insular Cortex
	1.55	34	22	-10	R Frontal Orbital Cortex
ET GPe FC – velocity of finger tapping ↑					
1296	3.30	-58	-44	46	L Supramarginal Gyrus, posterior division
	3.23	-56	-36	38	L Supramarginal Gyrus, anterior division
	3.13	-46	-34	16	L Parietal Operculum Cortex
	2.95	-40	-42	50	L Superior Parietal Lobule
	2.87	-66	-36	18	L Superior Temporal Gyrus, posterior division
	2.76	-40	-38	46	L Postcentral Gyrus
816	3.87	46	6	-2	R Insular Cortex
	3.55	30	2	8	R Putamen
	3.38	56	16	-2	R Inferior Frontal Gyrus, pars opercularis
	3.11	60	6	-10	R Superior Temporal Gyrus, anterior division
	2.65	54	22	-6	R Inferior Frontal Gyrus, pars triangularis
717	3.16	58	-30	36	R Supramarginal Gyrus, anterior division
	3.05	50	-28	8	R Planum Temporale
	3.03	64	-18	34	R Postcentral Gyrus
	3.00	66	-40	12	R Supramarginal Gyrus, posterior division
	2.62	58	-18	-6	R Superior Temporal Gyrus, posterior division
290	2.86	-14	-50	58	L Precuneous Cortex
	2.22	-14	-40	50	L Postcentral Gyrus
	2.19	-12	-26	38	L Cingulate Gyrus, posterior division
	1.96	-26	-48	64	L Superior Parietal Lobule
159	2.52	-36	46	28	L Frontal Pole
	2.36	-30	36	34	L Middle Frontal Gyrus
251	2.78	6	-8	58	R Juxtapositional Lobule Cortex (formerly Supplementary Motor Cortex)
	2.45	4	20	34	R Cingulate Gyrus, anterior division
	2.15	6	-16	58	R Precentral Gyrus
	2.05	8	12	44	R Paracingulate Gyrus
	1.99	-4	0	58	L Juxtapositional Lobule Cortex (formerly Supplementary Motor Cortex)
236	3.42	-54	-58	8	L Middle Temporal Gyrus, temporooccipital part
	3.10	-60	-66	10	L Lateral Occipital Cortex, inferior division
195	2.50	52	46	4	R Frontal Pole
	2.39	56	28	12	R Inferior Frontal Gyrus, pars triangularis
184	2.29	-46	18	-4	L Frontal Operculum Cortex
	2.07	-34	8	-14	L Insular Cortex
	1.95	-52	30	-10	L Frontal Orbital Cortex
183	3.16	-48	2	28	L Precentral Gyrus
	2.35	-46	12	28	L Inferior Frontal Gyrus, pars opercularis
180	2.59	36	48	34	R Frontal Pole
168	2.78	12	-44	-24	R Cerebellar Lobule I-IV
	2.71	-6	-40	-28	Brainstem
	2.30	-10	-40	-26	L Cerebellar Lobule I-IV
	2.21	-2	-58	-16	L Cerebellar Lobule V
136	3.20	-14	-72	-46	L Cerebellar Lobule VIIb
	3.07	-16	-74	-42	L Cerebellar Crus II
	3.00	-16	-70	-58	L Cerebellar Lobule VIIIa
	2.05	-20	-68	-30	L Cerebellar Lobule VI
130	2.71	36	-48	52	R Superior Parietal Lobule
	2.39	38	-42	38	R Supramarginal Gyrus, posterior division
	1.80	42	-34	48	R Postcentral Gyrus
119	3.19	50	-2	36	R Precentral Gyrus
	1.70	52	6	50	R Middle Frontal Gyrus
115	2.95	-26	-12	8	L Putamen
	2.48	-30	-4	2	L Caudate
	2.20	-34	-6	10	L Insular Cortex
71	2.05	-14	10	-2	L Caudate
	1.74	-20	10	-6	L Putamen
40	1.94	-20	6	10	L Putamen
ET GPe FC – velocity of finger tapping ↓					
140	2.40	10	58	2	R Frontal Pole
	2.37	8	54	6	R Paracingulate Gyrus
	2.18	4	42	8	R Cingulate Gyrus, anterior division
129	2.69	-8	44	30	L Superior Frontal Gyrus
	2.43	-6	58	32	L Frontal Pole
	1.89	-6	38	36	L Paracingulate Gyrus
100	2.35	-10	46	-6	L Paracingulate Gyrus
	2.19	-8	44	8	L Cingulate Gyrus, anterior division
	1.93	-4	30	-2	L Subcallosal Cortex
HS GPi FC – velocity of finger tapping ↑					
159	2.88	-36	10	0	L Insular Cortex
	2.60	-30	4	-2	L Putamen
	1.57	-16	0	-4	L Pallidum
110	2.86	-4	42	28	L Paracingulate Gyrus
	2.38	2	28	28	R Cingulate Gyrus, anterior division
	1.89	8	40	26	R Paracingulate Gyrus
ET GPi FC – velocity of finger tapping ↑					
365	3.15	32	22	-10	R Frontal Orbital Cortex
	2.84	40	22	-2	R Frontal Operculum Cortex
	2.61	56	10	-4	R Temporal Pole
	2.49	48	6	-2	R Central Opercular Cortex
	2.22	48	2	-10	R Planum Polare
	2.09	38	16	-10	R Insular Cortex
	1.88	60	10	-4	R Temporal Pole
326	2.88	-46	18	-4	L Frontal Operculum Cortex
	2.55	-52	30	-6	L Inferior Frontal Gyrus, pars triangularis
	2.53	-50	8	-4	L Temporal Pole
	2.50	-34	24	-6	L Frontal Orbital Cortex
	1.97	-56	14	0	L Inferior Frontal Gyrus, pars opercularis
	1.89	-34	18	-8	L Insular Cortex
185	3.03	26	10	2	R Putamen
	2.26	34	0	8	R Insular Cortex
122	2.76	40	-40	-22	R Temporal Occipital Fusiform Cortex
	2.57	46	-50	-36	R Cerebellar Crus I
	2.49	34	-44	-34	R Cerebellar Lobule VI
	2.16	38	-48	-42	R Cerebellar Crus II
119	2.70	-40	-38	46	L Postcentral Gyrus
	2.41	-32	-54	42	L Superior Parietal Lobule
	1.99	-44	-32	40	L Supramarginal Gyrus, anterior division
	1.94	-48	-44	54	L Supramarginal Gyrus, posterior division
116	3.37	30	18	60	R Middle Frontal Gyrus
	2.76	20	10	60	R Superior Frontal Gyrus
100	2.63	14	-32	-30	Brainstem
100	2.49	-10	-66	-16	L Cerebellar Lobule VI
	1.91	-22	-70	-30	L Cerebellar Crus I

In ET patients, dorsal DN-FC with the cerebellum (lobules I-IV and right lobule V), brainstem, BG, left occipital pole, parietal operculum cortex bilaterally, insular and temporal cortices as well as pre and postcentral gyri was positively correlated with finger tapping velocity.

No correlation was found between ventral DN- FC and finger tapping velocity neither in HS nor in ET patients (Fig. 2, Table 6).

#### GP

In HS, GPe-FC with left putamen and insular cortex was positively correlated with finger tapping velocity. Additionally, GPe-FC with left caudate, pallidum and thalamus, right putamen, hippocampus and amygdala, insular and temporal cortices and right precentral gyrus was negatively correlated with finger tapping velocity (Fig. 2, Table 6).

In ET patients, GPe-FC with several cerebellar regions (lobules I-IV, left lobules V, VI, VIIb, VIIIa and crus I), putamen, left caudate, insular cortex, temporo-parietal regions, supplementary motor area, pre and post-central gyri and frontal cortices was positively correlated with finger tapping velocity. Additionally, GPe-FC with frontal pole, cingulate and paracingulate gyri exhibited a negative correlation between with finger tapping velocity (Fig. 2, Table 6).

In HS, GPi-FC with left putamen and pallidum, paracingulate gyrus and left insular cortex was positively correlated with finger tapping velocity.

In ET patients, GPi-FC with the cerebellum (bilateral crus I, right crus II and lobule VI), right putamen, brainstem, insular and temporal cortices, left postcentral gyrus, right superior and middle frontal gyri was positively correlated with finger tapping velocity (Fig. 2, Table 6).

For ET patients, we also found significant correlations between dorsal and ventral DN and GPe and GPi-FC and essential tremor severity (FMTRS scores). Results are reported in the Supplementary Materials (Suppl. Fig. 3 and Suppl. Table 3).

## Discussion

The objective of this study was to explore the potential relationship between the structural and functional characteristics of the cerebellum and BG and objective measures of altered voluntary movement execution in individuals with ET. By doing so, we aimed to better understand the pathophysiology of altered movement execution in ET. Our findings confirm that individuals with ET exhibit altered execution of repetitive finger tapping, characterized by bradykinesia (slowness of movement), without the presence of other abnormalities [6], as identified through kinematic analysis. Our morphometric analysis did not reveal any significant changes in global, cerebellar, and pallidal volumes between individuals with ET and HS, accordingly with previous studies [51, 52]. Although some earlier studies have demonstrated white matter alterations in ET [53], a consistent reproducible pattern of atrophy has not been conclusively demonstrated in this condition [51, 52]. Interestingly, our data demonstrate a trend to volume reduction in bilateral GP of ET patients, in line with previous observations of bilateral GP iron deposition [54]. However, patients exhibited complex patterns of altered FC involving both the cerebellum and the BG. Interestingly, impaired movement execution, i.e., a lower movement velocity, was associated with decreased DN and GP-FC with cerebellar areas, striatum and sensorimotor areas.

### Slowed Voluntary Movement Execution in ET

The present study shows that ET patients perform sequential finger movement at lower speed that normal subjects. We have previously interpreted this finding as a consequence of a cerebellum involvement [2, 7, 42, 43], based on experimental observation demonstrating that the cerebellum encodes various kinematic parameters, including movement velocity, and that cerebellar diseases may be associated with movement slowness (bradykinesia) [7, 25].

### Increased Cerebellar Activity, and DN Motor Shift in ET

The first novel finding to be discussed is the evidence of increased activity of the cerebellar internal circuitry in ET patients, involving both anterior and posterior lobules. Also, we provided evidence of an altered FC between the cerebellum and various cortical areas, likely mediated by pathways that traverse the thalamic nuclei [18–21, 55]. In detail, we found that ET patients showed increased FC between both the dorsal and ventral DN and anterior and posterior cerebellar lobules. This finding is in line with existing evidence highlighting the involvement of both anterior and posterior cerebellar lobules in ET, as demonstrated by Buijink and colleagues [56]. ET patients demonstrated increased FC of dorsal DN with areas part of habitual motor network including thalamus, BG and bilateral somatosensory cortices, while demonstrating reduced functional connectivity with multimodal associative cortices. Intriguingly, a similar pattern is evidenced for the ventral portion of the DN, classically deputed to associative connections.

These findings are consistent with previous studies and support the notion of impaired connectivity between the cerebellum and multimodal associative areas, which may contribute to ET pathophysiology [16, 17, 57–59]. Moreover, these results evidence a shift of DN towards increased connectivity with motor network areas. This shift may reflect deregulated increased connectivity for dorsal DN, but also a neural plasticity adaptation concerning ventral DN connectivity, that in normal condition has a main associative role.

### Increased GPi Connectivity in ET

Equally important, our study highlights the significance of FC abnormalities of both the DN and the striatum as well as between the cerebellar hemispheres responsible for sensory-motor integration (specifically the antero-lateral hemispheric cerebellar lobules) and the GP [17]. The GPe, important relay nuclei in the indirect BG pathway, demonstrated increased connectivity with cerebellar areas and striatum, while demonstrating alternatively increased and decreased connectivity with multiple cortical areas. The GPi, serving as the primary output structure of the BG, demonstrated increased FC with components of habitual motor network such as cerebellar lobules, brainstem, striatum and somatosensory motor cortices, as well as different neocortical areas. The observation of enhanced FC of GP, especially within the habitual motor network, can be interpreted in two distinct ways. Firstly, it might indicate a primary pathological manifestation of altered GP activity, or it could potentially be a compensatory mechanism, in an effort to counterbalance the uncontrolled activation of the cerebellum [60–64]. Remarkably, high-frequency, desynchronized GP activity and increased connectivity with cerebellum and cortical motor areas have been observed in both patients and animal models of Parkinson's disease (PD) [60–64]. These findings have, in some instances, been associated with poorer motor performance or the progression of motor symptoms in patients. In ET patients augmented GP connectivity was associated with poorer cognitive performance [58, 65], but scarce evidences regard motor manifestation in ET and GP activity. The presence of similar abnormalities in GP connectivity patterns in ET patients raises intriguing questions regarding shared neurophysiological mechanisms between ET and PD, particularly concerning voluntary motor execution.

### Reduced Voluntary Movement Velocity in ET Associates with Weaker Motor Network FC

When considering the correlations between neuroimaging and kinematic data, our study revealed that decreased finger tapping velocity in ET patients was associated with weaker FC between both dorsal DN and GPi with the motor network encompassing the cerebellar (antero-lateral cerebellar hemispheres)-thalamo-striatal-sensorimotor cortex. GPe exhibited a peculiar relation with finger tapping velocity in ET. In fact while in HS GPe-FC within habitual motor network and temporo-insular cortices had mainly negative correlation with movement velocity, in ET GPe-FC with the same structures was demonstrated to be positive, involving also GPe-FC with cerebellar areas. Repetitive motor tasks, such as finger tapping, require precise and coordinated activation and inhibition of brain regions involved in motor control. Our findings underscore the significance of both cerebellar and BG activity in facilitating optimal motor performance. Proper activity in these structures enables enhanced movement velocity while minimizing reliance on higher cortical structures for movement preparation and planning. As a result, both cerebellar and BG facilitates efficient motor execution [26, 27]. Hence, we observed increased connectivity of GP and DN portions with cerebellar and motor network areas in ET patients and a positive correlation between the GP- and DN-FC and faster voluntary movement execution. This suggests a potential compensatory role of observed findings, in an attempt to counterbalance the reduced voluntary motor performance in ET patients. Adjunctively, connectivity outside habitual motor network, especially with temporo-insular areas, was correlated with better motor performance, thus reinforcing the hypothesis of a compensatory origin of the observed findings. Increased activity within the motor network as well as a recruitment of non-motor areas in an effort to improve motor performance can constitute a compensatory network, that in an overloading context can potentially lead to imbalances in global brain activity with detrimental effects.

Previous studies demonstrated altered FC in cerebellar and pallidal networks, also correlating with poorer motor and non-motor performances, but none of them focused on their role in altered voluntary movement execution in ET patients [15, 17, 55, 56, 58, 59, 65]. Dopamine transporter studies demonstrated reduced striatal dopaminergic uptake in certain subgroups of ET patients [66, 67], and our study recently correlated it with lower finger tapping velocity [33]. Dopaminergic dysfunction can lead to secondary changes in cerebellar activity, in order to sustain motor performance in striatum (putamen)-thalamus-M1 circuit hypofunction, as observed in PD [24]. The potential relationships between alterations in central dopaminergic tone and FC changes between the BG and cerebellum should be object of further exploration in future studies.

### Confounding Effect of Tremor and Potential Study Limitations

An important aspect to discuss is the potential confounding effect of tremor on movement related results. The present results extend observations by previous studies that alterations in FC within the cerebellar-thalamus-sensorimotor network is associated with the severity of tremor [17, 55]. More in detail, Tikoo et al demonstrated that tremor severity, tremor amplitude and peak frequency were significantly associated with altered FC of the dentate nucleus with cerebellar areas, sensorimotor cortex and thalamus/BG, respectively as confirmed by our supplementary analyses on clinical tremor severity. In particular, positive correlation with tremor severity included the FC of ventral DN with cerebellar and cortical areas, as well as the GPe-FC with other BG structures, while negative correlation involved both dorsal DN- and GPi-FC with widespread cortical areas. Hence, it could be argued that the results of the present study might reflect the severity of the tremor rather than the impairment of voluntary movement. Importantly, however, in our analysis, we have considered tremor as a covariate of non-interest, thus excluding the possibility that it may have influenced our results. Moreover, a recent study evidenced that movement velocity in ET is not influenced by tremor amplitude, and tremor does not correlate with subtle dopaminergic changes in ET [33]. Thus, we can infer that, like tremor, the underlying pathophysiological mechanisms of impaired movement execution in ET cannot be solely attributed to a singular anatomical structure. Rather, a complex interplay of partially overlapping brain areas contributes to the generation of both tremor and altered movement execution in ET.

This study has some limitations. Firstly, our sample size is relatively small, although it is consistent with the majority of fMRI studies on ET [16, 51], and is composed by a prevalence of men. It is important to consider that ET is a highly heterogeneous condition from a clinical perspective, and our study predominantly focused on patients with intermediate forms of the disease. Therefore, future research is needed to validate the findings of this study on larger and more diverse patient cohorts to further elucidate the potential role played by clinical heterogeneity. Moreover, we excluded patients presenting with ET soft signs, since, especially questionable dystonia, could influence kinematic measures, and alter functional connectivity [68, 69]. Another limitation is the absence of DAT imaging for clinical confirmation of the diagnosis. However, it is important to note that the clinical diagnosis of ET was based on the most recent diagnostic criteria, which do not require DAT imaging [1]. Finally, another potential limitation of the study is that we solely examined repetitive finger movements and did not assess movements of different types or involving other body segments. Consequently, the generalizability of our observations to other types of movements remains uncertain.

## Conclusions

Our study contributes to a better understanding of the pathophysiology of ET by highlighting the role of the cerebellum, BG and cerebral cortex within a dysfunctional network. In this regard, functional alterations in the networks may not only influences the generation of tremor but also contributes to impaired motor execution in ET. The results of this study could hold significance within the current research landscape, which is increasingly focused on identifying specific ET subtypes or even distinct individual diseases within the broader spectrum of ET. Such an approach would aid in early recognition of patients who may progress to develop parkinsonian syndromes and shed light on potential diverse underlying causes.

## Supplementary Information

Below is the link to the electronic supplementary material.

Supplementary file1 (DOCX 1298 KB)

## Data Availability

The data of this study are available from the corresponding author, M. Bologna, upon reasonable request.

## Abbreviations

BDI-II: Beck Depression Inventory
BET: Brain Extraction Tool
BG: Basal Ganglia
BOLD: Blood Oxygenation Level-Dependent
BTS: BTS Engineering
CSF: Cerebrospinal Fluid
CV: Coefficient of Variation
DN: Dentate Nucleus
ET: Essential Tremor
FAST: FMRIB Automated Segmentation Tool
FC: Functional Connectivity
FDR: False discovery rate
FIRST: FMRIB's Integrated Registration and Segmentation Tool
FLIRT: FMRIB's Linear Image Registration Tool
FNIRT: FMRIB's Non-Linear Image Registration Tool
FOV: Field of View
FSL: FMRIB's Software Library
FTMTRS: Fahn-Tolosa-Marin Tremor Rating Scale
GM: Grey Matter
GP: Globus Pallidus
GPi: Internal Globus Pallidus
GPe: External Globus Pallidus
GRMS^2: G root mean square squared
HS: Healthy Subjects
ICA-AROMA: Independent Component Analysis-Automatic Removal of Motion Artifacts
MDS-UPDRS-III: Movement Disorder Society - Unified Parkinson’s Disease Rating Scale, part III (motor section)
MNI: Montreal Neurological Institute
MoCA: Montreal Cognitive Assessment
MPRAGE: Magnetization-Prepared Rapid Gradient-Echo
MRI: Magnetic Resonance Imaging
PD: Parkinson's Disease
RMS: Root-Mean-Square
rs-fMRI: resting-state functional magnetic resonance imaging
SIENAX: Structural Imaging Evaluation of Normalized Atrophy
SMA: Supplementary motor area
SMART: System SMART motion
SPSS: Statistical Package for the Social Sciences
SUIT: Spatially Unbiased Infratentorial Template
WM: White Matter
