# Supplementary material for: The Role of Cerebellum and Basal Ganglia Functional Connectivity in Altered Voluntary Movement Execution in Essential Tremor
Source: Cerebellum. 2024 May 18;23(5):2060–81. doi: 10.1007/s12311-024-01699-6 (PMC11489212; doi:10.1007/s12311-024-01699-6)
Supplement: Supplementary file 1 — Supplementary file1 (DOCX 1298 KB) [file 12311_2024_1699_MOESM1_ESM.docx]

**Supplementary Materials**

**Supplementary Figure 1**: Maps of dorsal and ventral portions of the dentate nucleus (DN) rsFC in HS and in ET patients. The color bars represent *t* values. Red-yellow and blue-light blue areas indicate respectively regions positively and negatively correlated with dorsal and ventral DN. Statistical significance was considered at p<0.05, False Discovery Rate (FDR) corrected.


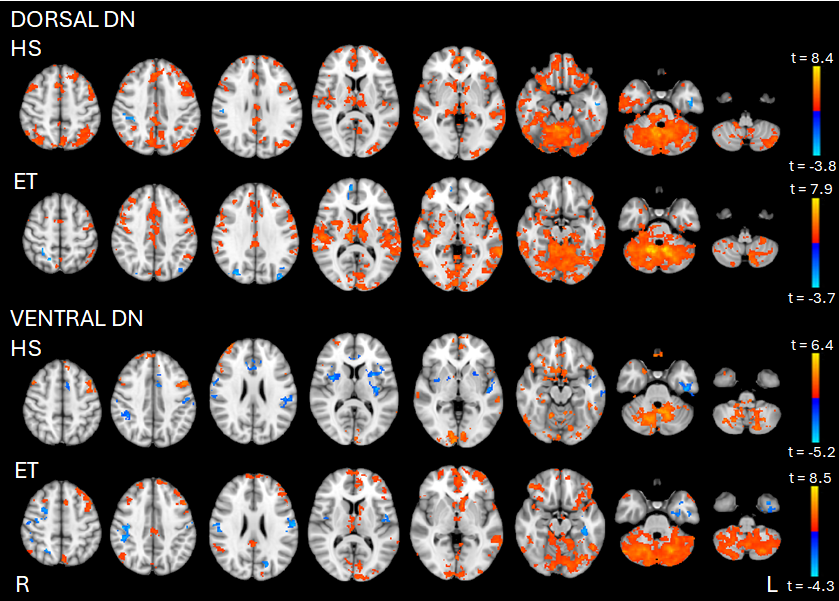


**Supplementary Table 1:** Dorsal and ventral DN rsFC in the single groups of HS and ET patients (one-sample t-test, p <0.05, false discovery rate corrected, minimum cluster extent set at 100 voxels). Positive and negative correlations in rsFC maps were reported. Anatomical localizations of peak MNI coordinates were established according to Harvard-Oxford cortical and subcortical structural atlases and the cerebellar atlas included in FMRIB’s Software Library.

|  |  | **MNI coordinates** | | |  |
| --- | --- | --- | --- | --- | --- |
| **Cluster size (voxels)** | **T** | **x** | **y** | **z** | **Cluster location (local maxima)** |
| **HS**  **Dorsal DN FC – positive** | | | | | |
| 29682 | 8.36 | 12 | -56 | -26 | R Cerebellar Lobule V |
|  | 6.90 | 10 | -50 | -22 | R Cerebellar Lobule I-IV |
|  | 6.41 | 26 | 12 | -16 | R Frontal Orbital Cortex |
|  | 6.24 | 4 | -60 | -30 | Vermis Lobule VIIIa |
|  | 6.04 | 4 | -60 | -26 | Vermis Lobule VI |
|  | 5.78 | 10 | -56 | -32 | R Cerebellar Lobule IX |
|  | 5.36 | -14 | -60 | -26 | L Cerebellar Lobule VI |
|  | 5.26 | -8 | -54 | -22 | L Cerebellar Lobule V |
| 664 | 3.42 | 50 | -58 | 52 | R Lateral Occipital Cortex, superior division |
|  | 3.18 | 38 | -52 | 54 | R Superior Parietal Lobule |
|  | 3.06 | 56 | -56 | 48 | R Angular Gyrus |
| 643 | 3.38 | 54 | 10 | 36 | R Precentral Gyrus |
|  | 3.33 | 52 | 30 | 24 | R Middle Frontal Gyrus |
|  | 3.01 | 48 | 12 | 24 | R Inferior Frontal Gyrus, pars opercularis |
|  | 2.90 | 54 | 36 | 14 | R Frontal Pole |
| 487 | 4.98 | 44 | -26 | 14 | R Heschl's Gyrus (includes H1 and H2) |
|  | 3.32 | 36 | -12 | 16 | R Insular Cortex |
|  | 3.32 | 54 | -20 | 6 | R Planum Temporale |
|  | 3.02 | 56 | -16 | 14 | R Central Opercular Cortex |
|  | 2.98 | 34 | -18 | -4 | R Putamen |
| 289 | 3.69 | 14 | 62 | 22 | R Frontal Pole |
| 167 | 3.27 | -2 | -20 | 26 | L Cingulate Gyrus, posterior division |
|  | 3.06 | 4 | -12 | 46 | R Cingulate Gyrus, anterior division |
|  | 2.12 | -2 | 0 | 48 | L Juxtapositional Lobule Cortex (formerly Supplementary Motor Cortex) |
| 137 | 3.69 | 58 | 12 | -6 | R Temporal Pole |
|  | 2.98 | 30 | 26 | -6 | R Frontal Orbital Cortex |
|  | 2.75 | 40 | 24 | 2 | R Frontal Operculum Cortex |
|  | 2.08 | 46 | 26 | 6 | R Inferior Frontal Gyrus, pars triangularis |
|  | 1.84 | 36 | 20 | 0 | R Insular Cortex |
| **HS**  **Dorsal DN FC – negative** | | | | | |
| 123 | 3.80 | -44 | -2 | -40 | L Inferior Temporal Gyrus, anterior division |
|  | 3.20 | -42 | -10 | -30 | L Inferior Temporal Gyrus, posterior division |
|  | 2.80 | -54 | -16 | -20 | L Middle Temporal Gyrus, posterior division |
|  | 2.27 | -48 | -8 | -22 | L Middle Temporal Gyrus, anterior division |
|  | 1.83 | -38 | 0 | -34 | L Temporal Fusiform Cortex, anterior division |
| 101 | 3.18 | 52 | -22 | 32 | R Supramarginal Gyrus, anterior division |
|  | 3.05 | 34 | -36 | 40 | R Postcentral Gyrus |
|  | 2.29 | 58 | -22 | 22 | R Parietal Operculum Cortex |
|  |  |  |  |  |  |
| **ET**  **Dorsal DN FC – positive** | | | | | |
| 33633 | 6.00 | -16 | -62 | -30 | L Cerebellar Lobule VI |
|  | 5.10 | 8 | -90 | -8 | R Occipital Pole |
|  | 4.98 | -66 | -48 | -2 | L Middle Temporal Gyrus, temporooccipital part |
|  | 4.94 | -24 | -70 | -28 | L Cerebellar Crus I |
|  | 4.89 | -24 | -70 | -40 | L Cerebellar Crus II |
|  | 4.87 | 14 | -18 | 8 | R Thalamus |
|  | 4.84 | 30 | -64 | -24 | R Cerebellar Lobule VI |
|  | 4.79 | -40 | -92 | -6 | L Occipital Pole |
|  | 4.60 | 12 | -50 | -20 | R Cerebellar Lobule V |
|  | 4.51 | 42 | -56 | -34 | R Cerebellar Crus I |
|  | 4.38 | -18 | 14 | 0 | L Putamen |
|  | 4.32 | 28 | -6 | 6 | R Putamen |
| 419 | 3.5 | -48 | -46 | 50 | L Supramarginal Gyrus |
|  | 3.29 | -48 | -60 | 48 | L Lateral Occipital Cortex, superior division |
|  | 3.29 | -38 | -50 | 60 | L Superior Parietal Lobule |
|  | 2.84 | -54 | -56 | 48 | L Angular Gyrus |
| 401 | 3.42 | 50 | 6 | 42 | R Precentral Gyrus |
|  | 2.69 | 48 | 12 | 36 | R Middle Frontal Gyrus |
|  | 2.33 | 42 | -18 | 38 | R Postcentral Gyrus |
| 252 | 3.57 | 0 | -60 | 62 | R Precuneous Cortex |
|  | 3.06 | -10 | -66 | 66 | L Lateral Occipital Cortex, superior division |
|  | 2.77 | -12 | -48 | 76 | L Postcentral Gyrus |
|  | 2.72 | -6 | -62 | 64 | L Precuneous Cortex |
| 223 | 4.35 | 46 | 50 | 8 | R Frontal Pole |
| 201 | 3.40 | 32 | 28 | 44 | R Middle Frontal Gyrus |
|  | 2.79 | 36 | 38 | 20 | R Frontal Pole |
| 176 | 3.70 | 28 | 38 | -12 | R Frontal Pole |
|  | 2.63 | 40 | 26 | -20 | R Frontal Orbital Cortex |
| 134 | 3.77 | -4 | -82 | 42 | L Precuneous Cortex |
|  | 3.16 | 4 | -78 | 46 | R Precuneous Cortex |
| 116 | 3.08 | 0 | 52 | -24 | R Frontal Medial Cortex |
|  | 2.81 | -14 | 58 | -16 | L Frontal Pole |
|  | 2.57 | -6 | 48 | -24 | L Frontal Medial Cortex |
|  | 2.20 | 4 | 58 | -24 | R Frontal Pole |
| 110 | 4.37 | -22 | -2 | -34 | L Parahippocampal Gyrus, anterior division |
|  | 2.60 | -32 | 2 | -44 | L Temporal Pole |
|  | 2.20 | -24 | 0 | -46 | L Temporal Fusiform Cortex, anterior division |
| **ET**  **Dorsal DN FC - negative** | | | | | |
| 182 | 3.48 | 8 | 60 | 18 | R Frontal Pole |
|  | 2.83 | 12 | 54 | 10 | R Paracingulate Gyrus |
|  | 2.29 | 12 | 52 | -4 | R Frontal Medial Cortex |
|  | 2.12 | 14 | 42 | 8 | R Cingulate Gyrus, anterior division |
| 128 | 3.13 | 30 | -78 | 32 | R Lateral Occipital Cortex, superior division |
| 126 | 3.62 | 22 | -52 | 56 | R Superior Parietal Lobule |
|  | 3.09 | 18 | -60 | 54 | R Lateral Occipital Cortex, superior division |
| 125 | 3.34 | -40 | -84 | 30 | L Lateral Occipital Cortex, superior division |
|  |  |  |  |  |  |
| **HS**  **Ventral DN FC – positive** | | | | | |
| 4682 | 6.34 | 18 | -68 | -40 | R Cerebellar Lobule VIIb |
|  | 5.04 | -16 | -62 | -32 | L Cerebella Lobule VI |
|  | 5.00 | -14 | -62 | -40 | L Cerebellar Lobule VIIIb |
|  | 4.61 | -26 | -70 | -42 | L Cerebellar Crus II |
|  | 4.53 | 4 | -50 | -24 | R Cerebellar Lobule I-IV |
|  | 4.46 | 14 | -56 | -42 | R Cerebellar Lobule IX |
|  | 4.39 | -4 | -54 | -28 | L Cerebellar Lobule I-IV |
|  | 4.39 | -14 | -66 | -44 | L Cerebellar Lobule VIIIa |
| 1782 | 4.49 | 16 | -94 | -12 | R Occipital Pole |
|  | 3.77 | 12 | -88 | 0 | R Intracalcarine Cortex |
|  | 3.70 | 32 | -76 | -22 | R Cerebellar Crus I |
|  | 3.56 | 4 | -88 | -16 | R Lingual Gyrus |
|  | 3.42 | 50 | -68 | -20 | R Lateral Occipital Cortex, inferior division |
|  | 3.38 | -8 | -84 | -2 | L Lingual Gyrus |
|  | 3.37 | -2 | -94 | 12 | L Occipital Pole |
|  | 3.36 | -24 | -84 | -20 | L Occipital Fusiform Gyrus |
| 991 | 4.32 | -6 | 30 | -28 | L Subcallosal Cortex |
|  | 3.83 | -4 | 38 | -14 | L Frontal Medial Cortex |
|  | 3.43 | 4 | 16 | -18 | R Subcallosal Cortex |
|  | 3.36 | 26 | 24 | -22 | R Frontal Orbital Cortex |
|  | 3.34 | 4 | 32 | -30 | R Frontal Medial Cortex |
|  | 3.05 | 46 | 20 | -24 | R Temporal Pole |
| 523 | 4.04 | -44 | 16 | 50 | L Middle Frontal Gyrus |
|  | 3.84 | -40 | -4 | 62 | L Precentral Gyrus |
|  | 2.58 | -54 | 14 | 28 | L Inferior Frontal Gyrus, pars opercularis |
| 503 | 3.36 | 54 | 36 | 18 | R Frontal Pole |
| 277 | 3.38 | 60 | 22 | 18 | R Inferior Frontal Gyrus, pars opercularis |
|  | 3.22 | 50 | 16 | 44 | R Middle Frontal Gyrus |
|  | 2.39 | 52 | 18 | 26 | R Inferior Frontal Gyrus, pars opercularis |
| 261 | 5.41 | -42 | 18 | -22 | L Temporal Pole |
|  | 4.61 | -36 | 20 | -20 | L Frontal Orbital Cortex |
| 230 | 4.79 | 24 | 6 | -38 | R Temporal Pole |
| 224 | 3.69 | -4 | 38 | 46 | L Superior Frontal Gyrus |
|  | 3.05 | 8 | 52 | 42 | R Frontal Pole |
|  | 2.43 | -8 | 32 | 36 | L Paracingulate Gyrus |
| 186 | 3.52 | -66 | -38 | 6 | L Superior Temporal Gyrus, posterior division |
|  | 3.20 | -66 | -50 | 0 | L Middle Temporal Gyrus, temporooccipital part |
| 157 | 3.96 | 38 | -32 | -26 | R Temporal Fusiform Cortex, posterior division |
|  | 3.29 | 26 | -34 | -18 | R Parahippocampal Gyrus |
| 126 | 4.26 | -8 | -34 | -38 | Brainstem |
| 120 | 3.52 | 64 | -30 | -8 | R Middle Temporal Gyrus, posterior division |
|  | 2.21 | 64 | -20 | -2 | R Superior Temporal Gyrus, posterior division |
| 105 | 3.40 | -12 | 50 | -8 | L Frontal Medial Cortex |
|  | 3.13 | 6 | 44 | 6 | R Cingulate Gyrus, anterior division |
|  | 2.60 | 2 | 48 | 2 | R Paracingulate Gyrus |
|  | 2.29 | -4 | 56 | -2 | L Frontal Pole |
| 101 | 2.55 | -6 | 16 | 68 | L Superior Frontal Gyrus |
|  |  |  |  |  |  |
| **HS**  **Ventral DN FC – negative** | | | | | |
| 445 | 3.87 | -46 | -18 | -30 | L Inferior Temporal Gyrus, posterior division |
|  | 3.80 | -42 | -20 | -30 | L Temporal Fusiform Cortex, posterior division |
|  | 3.76 | -46 | -8 | -18 | L Superior Temporal Gyrus, anterior division |
|  | 2.79 | -36 | -10 | -30 | L Temporal Fusiform Cortex, anterior division |
|  | 2.56 | -50 | -10 | -22 | L Middle Temporal Gyrus, anterior division |
| 398 | 5.19 | 6 | 26 | 18 | R Cingulate Gyrus, anterior division |
|  | 3.09 | -4 | 20 | 36 | L Paracingulate Gyrus |
|  | 3.04 | -4 | 32 | 16 | L Cingulate Gyrus, anterior division |
|  | 2.68 | 12 | 30 | 30 | R Paracingulate Gyrus |
| 379 | 4.09 | -30 | 8 | 10 | L Insular Cortex |
|  | 2.86 | -36 | -14 | 20 | L Central Opercular Cortex |
|  | 2.80 | -38 | 10 | 12 | L Frontal Operculum Cortex |
|  | 2.46 | -28 | -16 | 4 | L Putamen |
|  | 2.29 | -20 | 16 | 8 | L Caudate |
| 331 | 3.24 | -58 | -28 | 26 | L Supramarginal Gyrus, anterior division |
|  | 3.10 | -50 | -26 | 22 | L Parietal Operculum Cortex |
|  | 2.99 | -54 | -16 | 40 | L Postcentral Gyrus |
| 309 | 4.36 | 34 | 2 | 12 | R Insular Cortex |
|  | 2.60 | 30 | 0 | -4 | R Putamen |
|  | 2.54 | 44 | 8 | 14 | R Inferior Frontal Gyrus, pars opercularis |
|  | 2.22 | 42 | 6 | 8 | R Central Opercular Cortex |
|  | 2.05 | 20 | 0 | 2 | R Pallidum |
| 249 | 3.18 | 16 | -56 | 64 | R Superior Parietal Lobule |
|  | 3.10 | 12 | -60 | 66 | R Lateral Occipital Cortex, superior division |
|  | 3.10 | 20 | -42 | 74 | R Postcentral Gyrus |
|  | 2.76 | 12 | -54 | 56 | R Precuneous Cortex |
| 199 | 4.00 | 40 | -42 | 38 | R Supramarginal Gyrus, posterior division |
|  | 3.91 | 52 | -22 | 30 | R Supramarginal Gyrus, anterior division |
|  | 2.82 | 54 | -34 | 34 | R Parietal Operculum Cortex |
|  | 2.68 | 40 | -48 | 38 | R Angular Gyrus |
|  | 2.12 | 60 | -18 | 32 | R Postcentral Gyrus |
| 199 | 3.20 | 24 | 4 | 66 | R Superior Frontal Gyrus |
|  | 3.12 | 10 | -12 | 58 | R Juxtapositional Lobule Cortex (formerly Supplementary Motor Cortex) |
| 175 | 3.34 | -48 | -14 | -2 | L Planum Polare |
|  | 3.05 | -46 | -20 | 0 | L Heschl’s Gyrus (includes H1 and H2) |
|  | 2.48 | -52 | 0 | 2 | L Central Opercular Cortex |
|  | 2.28 | -50 | 4 | -14 | L Temporal Pole |
| 136 | 3.62 | 68 | -18 | 20 | R Postcentral Gyrus |
|  | 2.99 | 58 | 2 | 22 | R Precentral Gyrus |
|  | 2.51 | 60 | -24 | 24 | R Supramarginal Gyrus, anterior division |
|  | 2.25 | 52 | -22 | 12 | R Planum Temporale |
| 119 | 4.12 | -4 | 8 | 46 | L Paracingulate Gyrus |
|  | 3.65 | -10 | 4 | 46 | L Juxtapositional Lobule Cortex (formerly Supplementary Motor Cortex) |
|  | 2.93 | -8 | 4 | 34 | L Cingulate Gyrus, anterior division |
|  |  |  |  |  |  |
| **ET**  **Ventral DN FC – positive** | | | | | |
| 17719 | 8.21 | 14 | -70 | -36 | R Cerebellar Crus II |
|  | 7.03 | -20 | -66 | -34 | L Cerebellar Lobule VI |
|  | 6.75 | -20 | -68 | -38 | L Cerebellar Crus II |
|  | 5.81 | 20 | -66 | -32 | R Cerebellar Lobule VI |
|  | 5.30 | -14 | -66 | -38 | L Cerebellar Lobule VIIb |
|  | 5.29 | 32 | -70 | -36 | R Cerebellar Crus I |
| 3735 | 4.49 | -42 | 24 | 48 | L Middle Frontal Gyrus |
|  | 4.08 | -40 | 56 | 2 | L Frontal Pole |
|  | 4.03 | -8 | 10 | -6 | L Accumbens |
|  | 3.95 | -8 | 10 | -2 | L Caudate |
|  | 3.84 | -24 | 2 | -20 | L Amygdala |
|  | 3.68 | -32 | 16 | -14 | L Frontal Orbital Cortex |
| 2639 | 5.24 | 4 | 64 | 2 | R Frontal Pole |
|  | 4.25 | -6 | 62 | 4 | L Frontal Pole |
|  | 4.18 | 2 | 44 | 2 | R Cingulate Gyrus, anterior division |
|  | 4.16 | -2 | 44 | 2 | L Cingulate Gyrus, anterior division |
|  | 3.85 | -4 | 54 | 2 | L Paracingulate Gyrus |
|  | 3.82 | -10 | 50 | -8 | L Frontal Medial Cortex |
| 498 | 4.01 | -16 | 22 | 62 | L Superior Frontal Gyrus |
|  | 3.90 | 12 | 26 | 64 | R Superior Frontal Gyrus |
|  | 3.35 | -6 | 16 | 52 | L Paracingulate Gyrus |
| 346 | 3.93 | 42 | 10 | -16 | R Temporal Pole |
|  | 3.76 | 44 | 6 | -10 | R Insular Cortex |
|  | 3.36 | 34 | 26 | -22 | R Frontal Orbital Cortex |
|  | 3.22 | 42 | -10 | -8 | R Planum Polare |
| 221 | 3.48 | -8 | -36 | 32 | L Cingulate Gyrus, posterior division |
|  | 3.05 | 0 | -44 | 24 | R Cingulate Gyrus, posterior division |
| 213 | 3.96 | -2 | -70 | 56 | L Precuneous Cortex |
|  | 2.62 | -10 | -66 | 66 | L Lateral Occipital Cortex, superior division |
| 172 | 3.31 | -8 | -24 | 34 | L Cingulate Gyrus, posterior division |
| 170 | 3.31 | 46 | 24 | 44 | R Middle Frontal Gyrus |
|  | 2.56 | 24 | 18 | 50 | R Superior Frontal Gyrus |
| 149 | 2.62 | 48 | -64 | 48 | R Lateral Occipital Cortex, superior division |
|  | 1.74 | 52 | -54 | 36 | R Angular Gyrus |
| 142 | 3.11 | 56 | -52 | 18 | R Angular Gyrus |
|  | 2.56 | 54 | -44 | 20 | R Supramarginal Gyrus, posterior division |
| **ET**  **Ventral DN FC-negative** |  |  |  |  |  |
| 564 | 4.30 | 36 | -14 | 46 | R Precentral Gyrus |
|  | 3.78 | 36 | -22 | 44 | R Postcentral Gyrus |
|  | 3.40 | 48 | -34 | 44 | R Supramarginal Gyrus, anterior division |
|  | 3.19 | 48 | -38 | 44 | R Supramarginal Gyrus, posterior division |
|  | 3.14 | 42 | 4 | 56 | R Middle Frontal Gyrus |
|  | 2.73 | 24 | 4 | 48 | R Superior Frontal Gyrus |
| 373 | 3.71 | -44 | -8 | 14 | L Central Opercular Cortex |
|  | 3.30 | -60 | -14 | 26 | L Postcentral Gyrus |
|  | 2.44 | -52 | -2 | 28 | L Precentral Gyrus |
| 245 | 3.26 | 46 | -18 | 16 | R Central Opercular Cortex |
|  | 2.78 | 54 | -20 | 24 | R Postcentral Gyrus |
|  | 2.77 | 32 | -20 | 16 | R Insular Cortex |
| 226 | 3.29 | -36 | -30 | -20 | L Temporal Fusiform Cortex, posterior division |
|  | 2.80 | -38 | -22 | -14 | L Hippocampus |
|  | 1.84 | -46 | -38 | -18 | L Inferior Temporal Gyrus, posterior division |
| 195 | 2.80 | -38 | -4 | -44 | L Temporal Fusiform Cortex, anterior division |
|  | 2.71 | -32 | 4 | -48 | L Temporal Pole |
|  | 2.47 | -42 | -16 | -40 | L Inferior Temporal Gyrus, posterior division |
| 193 | 3.16 | 22 | -62 | 54 | R Lateral Occipital Cortex, superior division |
|  | 2.82 | 16 | -56 | 62 | R Superior Parietal Lobule |
|  | 2.75 | 12 | -42 | 62 | R Postcentral Gyrus |
| 152 | 3.27 | -10 | -20 | 64 | L Precentral Gyrus |
| 122 | 3.33 | -26 | -10 | -26 | L Hippocampus |
|  | 2.23 | -34 | -4 | -28 | L Parahippocampal Gyrus, anterior division |
| 105 | 3.14 | -20 | -78 | 26 | L Cuneal Cortex |
|  | 2.28 | -14 | -70 | 20 | L Precuneous Cortex |
|  | 2.07 | -16 | -68 | 16 | L Supracalcarine Cortex |

**Supplementary Figure 2**: Maps of external and internal segments of the globus pallidus (GPe and GPi) rsFC in HS and ET patients. The color bars represent *t* values. Red-yellow and blue-light blue areas indicate respectively regions positively and negatively correlated with GPe and GPi. Statistical significance was considered at p<0.05, False Discovery Rate (FDR) corrected.


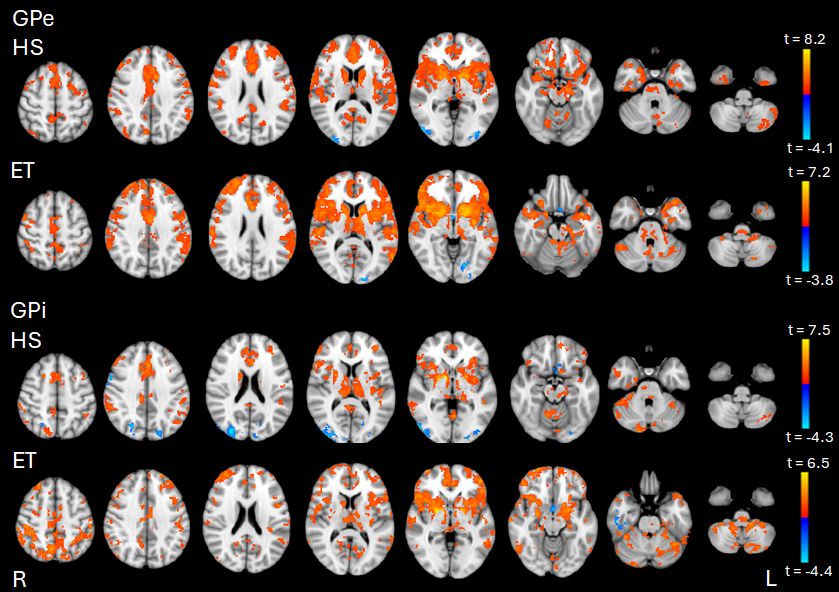


**Supplementary Table 2:** GPe and GPi rsFC in the single groups of HS and ET patients (one-sample t-test, p <0.05, false discovery rate corrected, minimum cluster extent set at 100 voxels). Positive and negative correlations in rsFC maps were reported. Anatomical localizations of peak MNI coordinates were established according to Harvard-Oxford cortical and subcortical structural atlases and the cerebellar atlas included in FMRIB’s Software Library.

|  |  | **MNI coordinates** | | |  |
| --- | --- | --- | --- | --- | --- |
| **Cluster size (voxels)** | **T** | **x** | **y** | **z** | **Cluster location (local maxima)** |
| **HS**  **GPe FC – positive** | | | | | |
| 32181 | 7.30 | -14 | 6 | -2 | L Pallidum |
|  | 6.90 | -8 | 4 | -2 | L Caudate |
|  | 6.25 | 18 | 2 | 0 | R Pallidum |
|  | 5.85 | -36 | 16 | -20 | R Frontal Orbital Cortex |
|  | 5.71 | -8 | 12 | 38 | L Paracingulate Gyrus |
|  | 5.64 | 4 | 26 | 20 | R Cingulate Gyrus, anterior division |
|  | 5.62 | 40 | 12 | -6 | R Insular Cortex |
|  | 5.47 | 20 | 8 | 2 | R Putamen |
| 1614 | 4.47 | -6 | -76 | -26 | L Cerebellar Lobule VI |
|  | 3.89 | -46 | -52 | -48 | L Cerebellar Crus II |
|  | 3.67 | -22 | -76 | -56 | L Cerebellar Lobule VIIb |
|  | 3.66 | -52 | -60 | -32 | L Cerebellar Crus I |
|  | 3.59 | -12 | -52 | -12 | L Cerebellar Lobule V |
|  | 3.42 | -26 | -64 | -56 | L Cerebellar Lobule VIIIa |
|  | 3.25 | -4 | -66 | -34 | Vermis Lobule VIIIa |
| 318 | 3.22 | 44 | -76 | 22 | R Lateral Occipital Cortex, superior division |
| 125 | 3.54 | 22 | -66 | -16 | R Cerebellar Lobule VI |
| **HS**  **GPe FC – negative** | | | | | |
| 266 | 4.02 | 50 | -78 | 0 | R Lateral Occipital Cortex, inferior division |
|  | 2.26 | 40 | -90 | 6 | R Occipital Pole |
| 142 | 3.74 | -34 | -86 | -4 | L Lateral Occipital Cortex, inferior division |
|  | 2.54 | -36 | -74 | -12 | L Occipital Fusiform Gyrus |
|  | 1.99 | -24 | -92 | -10 | L Occipital Pole |
| 108 | 3.41 | 26 | -98 | 8 | R Occipital Pole |
| **ET**  **GPe FC – positive** | | | | | |
| 39864 | 7.12 | -22 | 8 | -2 | L Putamen |
|  | 6.56 | 26 | 4 | -4 | R Putamen |
|  | 6.40 | 16 | 16 | -2 | R Caudate |
|  | 6.23 | 52 | 10 | 4 | R Inferior Frontal Gyrus, pars opercularis |
|  | 6.10 | 60 | 8 | 14 | R Precentral Gyrus |
|  | 5.98 | 34 | 16 | -6 | R Insular Cortex |
|  | 5.86 | -14 | 12 | -4 | L Caudate |
| 329 | 3.38 | 46 | -50 | -32 | R Cerebellar Crus I |
|  | 2.95 | 52 | -50 | -24 | R Inferior Temporal Gyrus, temporooccipital part |
|  | 2.40 | 46 | -60 | -20 | R Temporal Occipital Fusiform Cortex |
|  | 2.24 | 36 | -58 | -28 | R Cerebellar Lobule VI |
|  | 2.17 | 44 | -38 | -26 | R Temporal Fusiform Cortex, posterior division |
| 172 | 4.21 | 34 | -44 | -54 | R Cerebellar Lobule VIIIa |
|  | 2.56 | 20 | -46 | -56 | R Cerebellar Lobule VIIIb |
|  | 2.45 | 26 | -36 | -42 | R Cerebellar Lobule X |
|  | 2.43 | 32 | -36 | -40 | R Cerebellar Lobule VI |
| **ET**  **GPe FC - negative** | | | | | |
| 311 | 3.15 | -18 | -94 | 12 | L Occipital Pole |
|  | 3.03 | -16 | -76 | -4 | L Lingual Gyrus |
|  | 2.74 | -28 | -68 | -14 | L Occipital Fusiform Gyrus |
| 249 | 2.77 | 0 | 16 | -6 | R Subcallosal Cortex |
|  | 2.36 | -4 | 12 | -20 | L Subcallosal Cortex |
|  | 2.24 | -10 | 18 | -26 | L Frontal Orbital Cortex |
| **HS**  **GPi FC – positive** | | | | | |
| 10654 | 7.39 | 12 | 0 | -2 | R Pallidum |
|  | 7.26 | 0 | 26 | 24 | R Cingulate Gyrus |
|  | 5.41 | 34 | 14 | -6 | R Insular Cortex |
|  | 5.33 | -16 | -18 | 8 | L Thalamus |
|  | 5.00 | -22 | -6 | -10 | L Amygdala |
|  | 4.91 | -40 | 50 | 8 | L Frontal Pole |
|  | 4.85 | 6 | 12 | 36 | R Cingulate Gyrus, anterior division |
|  | 4.75 | 24 | 12 | 6 | R Putamen |
| 911 | 4.39 | -30 | -22 | 8 | L Putamen |
|  | 4.08 | -50 | -42 | 16 | L Planum Temporale |
|  | 3.78 | -46 | -40 | 8 | L Superior temporal Gyrus, posterior division |
|  | 3.61 | -52 | -12 | 18 | L Central Opercular Cortex |
|  | 3.60 | -34 | -28 | 8 | L Heschl's Gyrus (includes H1 and H2) |
|  | 3.22 | -52 | -34 | 36 | L Supramarginal Gyrus, anterior division |
|  | 3.21 | -48 | -40 | -2 | L Middle Temporal Gyrus, posterior division |
|  | 3.16 | -56 | -16 | 28 | L Postcentral Gyrus |
|  | 3.13 | -34 | -24 | 12 | L Insular Cortex |
|  | 3.06 | -64 | -44 | 34 | L Supramarginal Gyrus, posterior division |
| 596 | 3.80 | 58 | -34 | 52 | R Supramarginal Gyrus, posterior division |
|  | 3.46 | 64 | -26 | 44 | R Supramarginal Gyrus, anterior division |
|  | 2.93 | 48 | -56 | 46 | R Angular Gyrus |
|  | 2.79 | 38 | -50 | 42 | R Superior Parietal Lobule |
| 470 | 3.83 | 12 | -28 | 36 | R Cingulate Gyrus, posterior division |
|  | 2.89 | 10 | -42 | 48 | R Precuneous Cortex |
|  | 2.78 | 4 | -12 | 28 | R Cingulate Gyrus, anterior division |
| 356 | 3.90 | 44 | -46 | -34 | R Cerebellar Crus I |
|  | 3.64 | 28 | -34 | -28 | R Cerebellar Lobule V |
|  | 2.93 | 38 | -56 | -24 | R Cerebellar Lobule VI |
| 254 | 3.54 | -24 | -74 | -22 | L Cerebellar Lobule VI |
|  | 3.26 | -20 | -70 | -34 | L Cerebellar Crus I |
|  | 3.09 | -24 | -74 | -44 | L Cerebellar Crus II |
| 209 | 3.21 | -22 | 12 | 58 | L Superior Frontal Gyrus |
|  | 3.05 | -34 | 10 | 62 | L Middle Frontal Gyrus |
| 185 | 3.38 | 62 | -50 | -4 | R Middle Temporal Gyrus, temporooccipital part |
|  | 2.64 | 58 | -58 | -12 | R Inferior Temporal Gyrus, temporooccipital part |
|  | 2.50 | 66 | -34 | 2 | R Superior Temporal Gyrus, posterior division |
| 177 | 3.06 | 12 | -74 | 58 | R Lateral Occipital Cortex, superior division |
|  | 2.30 | 14 | -74 | 40 | R Precuneous Cortex |
| 150 | 3.49 | -4 | -82 | -16 | L Lingual Gyrus |
|  | 2.72 | -10 | -78 | -32 | L Cerebellar Crus II |
|  | 2.65 | -8 | -78 | -28 | L Cerebellar Crus I |
|  | 2.20 | 6 | -76 | -20 | Vermis Lobule VI |
|  | 1.95 | 0 | -78 | -34 | Vermis Crus II |
|  | 1.85 | -12 | -74 | -42 | L Cerebellar Lobule VIIb |
| 150 | 4.15 | 16 | -92 | -30 | R Cerebellar Crus II |
|  | 4.13 | 20 | -86 | -26 | R Cerebellar Crus I |
| 144 | 3.32 | -32 | -50 | -32 | L Cerebellar Lobule VI |
|  | 3.12 | -40 | -62 | -24 | L Cerebellar Crus I |
| 125 | 3.28 | 52 | -4 | -28 | R Middle Temporal Gyrus, anterior division |
|  | 2.88 | 54 | 10 | -24 | R Temporal Pole |
|  | 2.63 | 58 | -6 | -36 | R Inferior Temporal Gyrus, anterior division |
| 121 | 3.38 | 50 | 28 | 36 | R Middle Frontal Gyrus |
|  | 2.64 | 38 | 36 | 34 | R Frontal Pole |
| 111 | 3.14 | -40 | 16 | 56 | L Middle Frontal Gyrus |
| 103 | 3.19 | -44 | -46 | 50 | L Supramarginal Gyrus, posterior division |
|  | 2.76 | -44 | -52 | 54 | L Angular Gyrus |
| **HS**  **GPi FC – negative** | | | | | |
| 1043 | 4.23 | 42 | -64 | -10 | R Lateral Occipital Cortex, inferior division |
|  | 4.15 | 30 | -84 | 20 | R Lateral Occipital Cortex, superior division |
|  | 3.36 | 36 | -90 | 14 | R Occipital Pole |
| 497 | 3.96 | 12 | -40 | 72 | R Postcentral Gyrus |
|  | 3.35 | 22 | -60 | 58 | R Lateral Occipital Cortex, superior division |
|  | 3.01 | 20 | -56 | 56 | R Superior Parietal Lobule |
| 349 | 3.11 | -20 | -84 | 36 | L Lateral Occipital Cortex, superior division |
|  | 2.93 | -18 | -70 | 16 | L Cuneal Cortex |
|  | 2.71 | -10 | -96 | 18 | L Occipital Pole |
| 275 | 3.47 | -38 | -82 | 2 | L Lateral Occipital Cortex, inferior division |
|  | 3.28 | -26 | -80 | -10 | L Occipital Fusiform Gyrus |
|  | 2.52 | -24 | -92 | -2 | L Occipital Pole |
|  | 2.44 | -16 | -78 | -12 | L Lingual Gyrus |
| 155 | 3.17 | -10 | 18 | -26 | L Frontal Orbital Cortex |
|  | 2.82 | -10 | 18 | -22 | L Subcallosal Cortex |
|  | 2.77 | -12 | 28 | -22 | L Frontal Orbital Cortex |
|  | 2.60 | 2 | 16 | -16 | R Subcallosal Cortex |
| 141 | 3.99 | 24 | -72 | 36 | R Occipital Cortex, superior division |
| 128 | 3.48 | 58 | 2 | 30 | R Precentral Gyrus |
|  | 2.56 | 62 | -8 | 30 | R Postcentral Gyrus |
|  |  |  |  |  |  |
| **ET**  **GPi FC – positive** | | | | | |
| 27413 | 6.43 | 22 | -2 | 0 | R Pallidum |
|  | 6.21 | -18 | -2 | -4 | L Pallidum |
|  | 5.76 | 24 | 4 | -4 | R Putamen |
|  | 5.13 | 8 | -62 | 62 | R Precuneous Cortex |
|  | 5.08 | 10 | -62 | 66 | R Lateral Occipital Cortex, superior division |
|  | 5.08 | -24 | 2 | -10 | L Putamen |
|  | 4.99 | 10 | -26 | -34 | Brainstem |
|  | 4.95 | 10 | -2 | -2 | R Thalamus |
|  | 4.90 | 26 | -8 | -10 | R Amygdala |
|  | 4.85 | 26 | -42 | 60 | R Superior Parietal Lobule |
|  | 4.64 | -36 | -44 | -42 | L Cerebellar Crus II |
| 577 | 3.68 | -50 | 4 | 20 | L Precentral Gyrus |
|  | 3.03 | -50 | 12 | 26 | L Inferior Frontal Gyrus, pars opercularis |
|  | 3.02 | -38 | 16 | 40 | L Middle Frontal Gyrus |
| 490 | 3.58 | 2 | -58 | 4 | R Lingual Gyrus |
|  | 3.51 | -8 | -60 | 12 | L Precuneous Cortex |
|  | 3.02 | -18 | -70 | 10 | L Intracalcarine Cortex |
|  | 2.65 | 2 | -56 | 8 | R Precuneous Cortex |
|  | 2.33 | -20 | -70 | 22 | L Cuneal Cortex |
|  | 2.28 | -6 | -60 | 2 | L Lingual Gyrus |
|  | 2.27 | 14 | -70 | 14 | R Intracalcarine Cortex |
| 424 | 3.57 | 56 | -58 | -12 | R Inferior Temporal Gyrus, temporooccipital part |
|  | 3.22 | 50 | -70 | -30 | R Cerebellar Crus I |
|  | 3.01 | 64 | -58 | -6 | R Middle Temporal Gyrus, temporooccipital part |
|  | 2.29 | 48 | -62 | -18 | R Lateral Occipital Cortex, inferior division |
|  | 2.17 | 42 | -66 | -16 | R Occipital Fusiform Gyrus |
| 381 | 4.03 | 10 | -46 | -50 | R Cerebellar Lobule IX |
|  | 3.71 | 18 | -50 | -58 | R Cerebellar Lobule VIIIb |
|  | 3.48 | 4 | -44 | -56 | Brainstem |
|  | 2.97 | 14 | -40 | -46 | R Cerebellar Lobule X |
| 229 | 3.85 | -54 | -14 | -8 | L Superior Temporal Gyrus, posterior division |
|  | 3.31 | -58 | -14 | -10 | L Middle Temporal Gyrus, posterior division |
|  | 2.90 | -50 | -8 | 0 | L Planum Polare |
|  | 2.66 | -48 | -4 | 6 | L Central Opercular Cortex |
|  | 2.48 | -56 | -14 | 2 | L Planum Temporale |
|  | 2.38 | -56 | -12 | 6 | L Heschl's Gyrus (includes H1 and H2) |
| 138 | 3.00 | 28 | -84 | -22 | R Cerebellar Crus I |
|  | 2.04 | 16 | -92 | -28 | R Cerebellar Crus II |
| 137 | 3.32 | -14 | 58 | -10 | L Frontal Pole |
|  | 2.16 | -4 | 52 | -12 | L Frontal Medial Cortex |
| 134 | 3.07 | 46 | -72 | 32 | R lateral Occipital Cortex, superior division |
| 114 | 3.52 | 8 | 26 | 48 | R Superior Frontal Gyrus |
|  | 2.72 | 8 | 30 | 28 | R Paracingulate Gyrus |
| 103 | 2.80 | -56 | -48 | -30 | L Inferior Temporal Gyrus, temporooccipital part |
|  | 2.61 | -62 | -38 | -24 | L Inferior Temporal Gyrus, posterior division |
|  |  |  |  |  |  |
| **ET**  **GPi FC-negative** |  |  |  |  |  |
| 152 | 3.54 | 34 | -22 | -30 | R Temporal Fusiform Cortex, posterior division |
|  | 3.47 | 52 | -20 | -26 | R Inferior Temporal Gyrus, posterior division |
|  | 3.05 | 52 | -4 | -26 | R Middle Temporal Gyrus, anterior division |
|  | 2.99 | 56 | -10 | -20 | R Middle Temporal Gyrus, posterior division |

*Correlation of FC with clinical scores.*

*Methods*

In patients’ group, voxel-wise correlations between either dorsal and ventral portions of the DN or external and internal segments of the GP FC maps and clinical scores (FMTRS scores) were non parametrically performed (FSL randomise, 5000 permutations), with age and gender as covariates of no interest. Results were corrected using false discovery rate (FDR) correction [Benjamini et al. 1995] for multiple comparisons (p < 0.05). The minimum cluster extent was set at 100 voxels.

*Results*

*DN*

In ET patients, dorsal DN-FC with the left inferior and middle temporal gyri exhibited a positive correlation with tremor severity (Suppl. Fig. 3, Suppl. Table 3), while dorsal DN-FC with cingulate and paracingulate cortices and occipital fusiform, lingual and intracalcarine regions showed a negative correlation with tremor severity (Suppl. Fig. 3, Suppl. Table 3). Ventral DN-FC with the cerebellum (right lobule VI, crus I and II), bilateral frontal pole and paracingulate cortex was positively correlated with tremor severity (Suppl. Fig. 3, Suppl. Table 3).

*GP*

In ET patients, GPe-FC with the left putamen, pallidum and insular cortex showed a positive correlation with tremor severity, while GPe-FC with pre and post central gyri, anterior cingulate cortex, inferior and middle fontal gyri and orbitofrontal regions exhibited a negative correlation with tremor severity (Suppl. Fig. 3, Suppl. Table 3). Finally, GPi-FC with postcentral gyri, precuneus, left superior parietal lobule and angular gyrus exhibited a negative correlation with tremor severity (Supplementary Fig. 3).

**Supplementary Figure 3**: Correlation maps of dorsal and ventral portions of the dentate nucleus (DN) rsFC and external and internal segments of the globus pallidus (GPe and GPi) rsFC with tremor severity in ET patients. Positive correlations are shown in red-yellow color, and negative correlations are shown in blue-light blue color. Statistical significance was considered at p < 0.05 (FDR corrected).

**
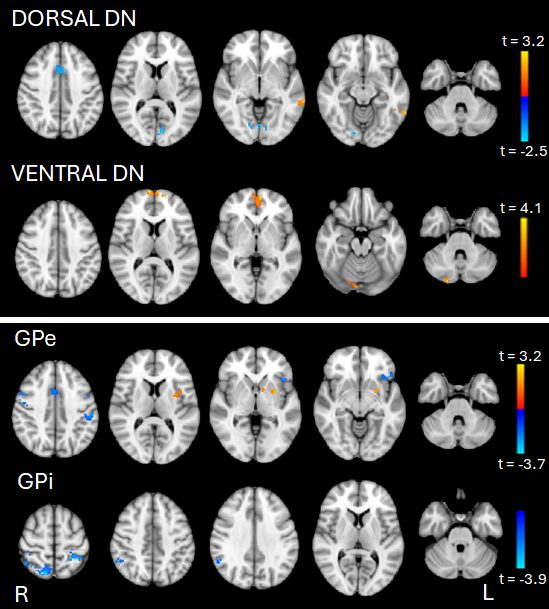
**

**Supplementary Table 3:** Brain regions showing significant correlations between dorsal and ventral portions of the dentate nucleus (DN) and external and internal segments of the globus pallidus (GPe and GPi) functional connectivity maps and tremor severity in ET patients (p <0.05, false discovery rate corrected, minimum cluster extent set at 100 voxels). Anatomical localizations of peak MNI coordinates were established according to Harvard-Oxford cortical and subcortical structural atlases and the cerebellar atlas included in FMRIB’s Software Library.

|  |  | **MNI coordinates** | | |  |
| --- | --- | --- | --- | --- | --- |
| **Cluster size (voxels)** | **T** | **x** | **y** | **z** | **Cluster location (local maxima)** |
| **ET**  **Dorsal DN FC – tremor severity ↑** | | | | | |
| 154 | 3.19 | -54 | -46 | -16 | L Inferior Temporal Gyrus, temporooccipital part |
|  | 2.71 | -68 | -36 | -6 | L Middle Temporal Gyrus, posterior division |
| **ET**  **Dorsal DN FC – tremor severity ↓** | | | | | |
| 170 | 2.44 | -2 | -78 | -2 | L Lingual Gyrus |
|  | 2.30 | 10 | -76 | 4 | R Intracalcarine Cortex |
|  | 2.21 | -8 | -82 | 10 | L Intracalcarine Cortex |
|  | 2.11 | 16 | -70 | 0 | R Lingual Gyrus |
|  | 2.02 | 18 | -82 | -12 | R Occipital Fusiform Gyrus |
| 165 | 2.17 | -4 | 16 | 34 | L Cingulate Gyrus, anterior division |
|  | 1.98 | 0 | 6 | 34 | R Cingulate Gyrus, anterior division |
|  | 1.86 | 2 | 14 | 42 | R Paracingulate Gyrus |
| **ET**  **Ventral DN FC – tremor severity ↑** | | | | | |
| 387 | 4.01 | 8 | 68 | 10 | R Frontal Pole |
|  | 3.89 | -6 | 58 | 0 | L Frontal Pole |
|  | 2.33 | -6 | 54 | -12 | L Frontal Medial Cortex |
|  | 2.23 | -6 | 42 | -2 | L Paracingulate Gyrus |
| 121 | 3.27 | 20 | -90 | -34 | R Cerebellar Crus II |
|  | 2.85 | 26 | -82 | -24 | R Cerebellar Crus I |
|  | 2.57 | 8 | -84 | -18 | R Lingual Gyrus |
|  | 1.70 | 20 | -76 | -20 | R Cerebellar Lobule VI |
| **ET**  **GPe FC – tremor severity ↑** | | | | | |
| 255 | 3.19 | -28 | 4 | 2 | L Putamen |
|  | 2.07 | -40 | 8 | 12 | L Central Opercular Cortex |
|  | 2.01 | -36 | -2 | 8 | L Insular Cortex |
|  | 1.91 | -16 | 2 | -6 | L Pallidum |
|  | 1.66 | -26 | 0 | -14 | L Amygdala |
| **ET**  **GPe FC – tremor severity ↓** | | | | | |
| 238 | 3.67 | 38 | -12 | 66 | R Precentral Gyrus |
|  | 2.09 | 52 | 6 | 50 | R Middle Frontal Gyrus |
| 131 | 2.34 | -42 | 22 | 0 | L Frontal Operculum Cortex |
|  | 2.08 | -38 | 22 | -4 | L Frontal Orbital Cortex |
|  | 1.57 | -54 | 22 | 6 | L Inferior Frontal Gyrus, pars triangularis |
|  | 1.48 | -52 | 18 | -2 | L Inferior Frontal Gyrus, pars opercularis |
|  | 1.43 | -32 | 22 | 2 | L Insular Cortex |
| 128 | 2.39 | -2 | 2 | 28 | L Cingulate Gyrus, anterior division |
| 127 | 2.66 | -54 | -26 | 42 | L Postcentral Gyrus |
|  | 2.33 | -54 | -32 | 42 | L Supramarginal Gyrus, anterior division |
| 124 | 2.59 | -40 | -48 | 60 | L Superior Parietal Lobule |
| 101 | 2.62 | -52 | -2 | 30 | L Precentral Gyrus |
| **ET**  **GPi FC – tremor severity ↓** | | | | | |
| 585 | 3.86 | 14 | -68 | 58 | R Lateral Occipital Cortex, superior division |
|  | 2.72 | 42 | -54 | 54 | R Angular Gyrus |
|  | 2.71 | 40 | -38 | 56 | R Superior Parietal Lobule |
|  | 2.53 | 8 | -46 | 68 | R Precuneous Cortex |
|  | 2.50 | 18 | -42 | 72 | R Postcentral Gyrus |
| 360 | 2.80 | -24 | -46 | 58 | L Superior Parietal Lobule |
|  | 2.41 | -26 | -62 | 64 | L Lateral Occipital Cortex, superior division |
|  | 2.40 | -22 | -34 | 68 | L Postcentral Gyrus |
|  | 2.38 | -10 | -62 | 50 | L Precuneous Cortex |
| 144 | 3.03 | 60 | -52 | 44 | R Angular Gyrus |

Benjamini Y, Hochberg Y (1995) Controlling the false discovery rate: a practical and powerful approach to multiple testing. J Roy Stat Soc: Ser B (Methodol) 57:289–300. https:// doi. org/ 10. 1111/j. 2517- 6161.995. tb020 31.x
